# A Markovian dynamics for *Caenorhabditis elegans* behavior across scales

**DOI:** 10.1073/pnas.2318805121

**Published:** 2024-07-31

**Authors:** Antonio C. Costa, Tosif Ahamed, David Jordan, Greg J. Stephens

**Affiliations:** ^a^Department of Physics and Astronomy, Vrije Universiteit Amsterdam, Amsterdam 1081HV, The Netherlands; ^b^Janelia Research Campus, HHMI, Ashburn, VA 20147; ^c^Department of Biochemistry, University of Cambridge, Cambridge CB2 1GA, United Kingdom; ^d^Biological Physics Theory Unit, Okinawa Institute of Science and Technology Graduate University, Okinawa 904-0495, Japan

**Keywords:** locomotion, animal behavior, coarse-graining, statistical physics, nonlinear dynamics

## Abstract

How do we capture the breadth of behavior in animal movement, from rapid body twitches to aging? Using high-resolution videos of the nematode worm *Caenorhabditis elegans*, we show that a single dynamics connects posture-scale fluctuations with trajectory diffusion and longer-lived behavioral states. We take short posture sequences as an instantaneous behavioral measure, fixing the sequence length for maximal prediction. Within the space of posture sequences, we construct a fine-scale, maximum entropy partition so that transitions among microstates define a high-fidelity Markov model, which we also use as a means of principled coarse-graining. We translate these dynamics into movement using resistive force theory, capturing the statistical properties of foraging trajectories. Predictive across scales, we leverage the longest-lived eigenvectors of the inferred Markov chain to perform a top–down subdivision of the worm’s foraging behavior, revealing both “runs-and-pirouettes” as well as previously uncharacterized finer-scale behaviors. We use our model to investigate the relevance of these fine-scale behaviors for foraging success, recovering a trade-off between local and global search strategies.

## Significance

Complex phenotypes, such as an animal’s behavior, generally depend on an overwhelming number of processes that span a vast range of scales. While there is no reason that behavioral dynamics permit simple models, by subsuming inherent nonlinearities and memory into maximally predictive microstates, we find one for *Caenorhabditis elegans* foraging. The resulting “Markov worm” is effectively indistinguishable from real worm motion across a range of timescales, and we can decompose our model dynamics both to recover and reveal behavioral states. Finally, we connect postures to trajectories, illuminating how worms explore the environment in different behavioral states.

From molecular motors contracting muscles, to neurons processing an ever changing environment, or the large-scale diffusion of hormones and other neuromodulatory chemicals, animal behavior arises from biological activity across innumerable spatial and temporal scales. With an instantaneous snapshot of all of these variables, the future behavioral state of the animal would be uniquely defined, a biological setting for the demon of Laplace (see e.g., ref. 1). Of course, such an approach is practically unrealizable. We are limited to a much smaller set of observations and the unobserved degrees of freedom will generally induce non-Markovianity, or memory, to the dynamics of the variables that we do measure (2, 3). In animal behavior, interpretations of this memory guide our understanding of the complexity of the process (4, 5). But what if we could use our observations to construct memory-full state variables that admit predictive, yet minimal-memory dynamics (6, 7)?

The construction of such dynamics appears daunting. We may even conclude that this is impossible if it were not for the fact that it is done routinely in physical systems. Indeed, it is often the case that a subset of observable functions is enough to capture behavior at a particular scale. Hydrodynamics, for example, can be formulated with effective variables such as fluid velocity, density, or temperature: their memory only coming from the previous state. In behavior, we expect the emergent reconstructed dynamics to be generally high-dimensional in order to account for the multitude of unobserved mechanisms. Yet, our approach also suggests a principled coarse-graining. Since the dynamics of the reconstructed states are Markovian, the emergent timescales of the (nonlinear) system are naturally ordered by the eigenvalue spectrum of a *linear* evolution operator, or transition matrix in the case of discrete states. The eigenvectors associated with the gaps in the spectrum indicate slow collective modes and provide natural targets for coarse-graining. In the hydrodynamic example of ∼10^23^ interacting molecules, these modes are the effective variables.

Here, we seek such Markov dynamics from the time series of posture in the foraging behavior of the nematode worm *Caenorhabditis elegans*, an important model organism in genetics and neuroscience (8–10). For both, the worm and animals generally, the collection of high-resolution behavioral data has been greatly accelerated by advancing techniques for pose estimation via machine vision (11–15), combined with computational and imaging improvements. Such measurement advances demand new behavioral understanding: analyses, models, and theory of posture-scale dynamics (4, 16, 17).

We implement a principled, generally applicable framework which combines delay embedding with Markov modeling (6). In this approach, we seek to overcome the partial observability of behavioral dynamics; variables which influence behavior but are instantaneously hidden become apparent over time and Markov predictability provides the quantitative measure of a self-determined system. Posture itself is a very complicated function of its underlying biological variables. In such situations, an initial expansion of dimensionality can simplify computations like function estimation and classification. We thus trade the complex modeling of a low-dimensional time series for the simpler modeling of a much higher-dimensional state space: The encoding of the unobserved degrees of freedom through time delays drastically simplifies our theory, leading to a powerful yet simple description of the emergent nonlinear Markovian dynamics.

While Markov approaches have an extensive history, perhaps most familiarly in Markov Chain Monte Carlo sampling of equilibrium distributions in “statistical physics” (18), substantially less attention has focused on a Markov encoding of actual dynamics, especially with a large number of states. Importantly, we note that there is no guarantee that our approach will work; for example, the number of necessary delays may be computationally prohibitive. But even this “failure” would provide important information about the memory of the system. On the other hand, if we are successful, we will be left with a finite set of observables that are approximately self-determined, measurable, and whose dynamics span the timescales that are relevant to the phenomena of interest. Such observables are likely to be biologically meaningful.

We find state variables for worm behavior that exhibit Markovian evolution across the multiple timescales of *C. elegans* foraging behavior: from fine-scale posture movements to “run-and-pirouette” strategies. Additionally, the macroscopic variables we reveal are not some baroque non-physical mathematical functions but rather correspond to interpretable behavioral motifs. We rediscover canonical behaviors from the rich history of *C. elegans* ethomics, as well as describe new ones. Each of these motifs is associated with its own characteristic timescale, and with them, we provide a new hierarchical subdivision of behavior. We show how the dynamics of these macroscopic variables can be propagated through a model of the organisms physical interaction with the environment to accurately predict locomotion from posture. Finally, we dissect the function of these behavioral motifs by investigating their relation to the exploration and exploitation of food sources.

## Short-Time Behaviors As Maximally Predictive Posture Sequences

On a 2D agar plate, worms move by making dorsoventral bends along their bodies (19). At the shortest timescales (∼1 s), these traveling waves along the body give rise to forward, backward, and turning locomotion (20–22). We show here that this organization emerges naturally from short posture sequences, formally a delay embedding first introduced in the theory of dynamical systems (23–27).

**Fig. 1.**
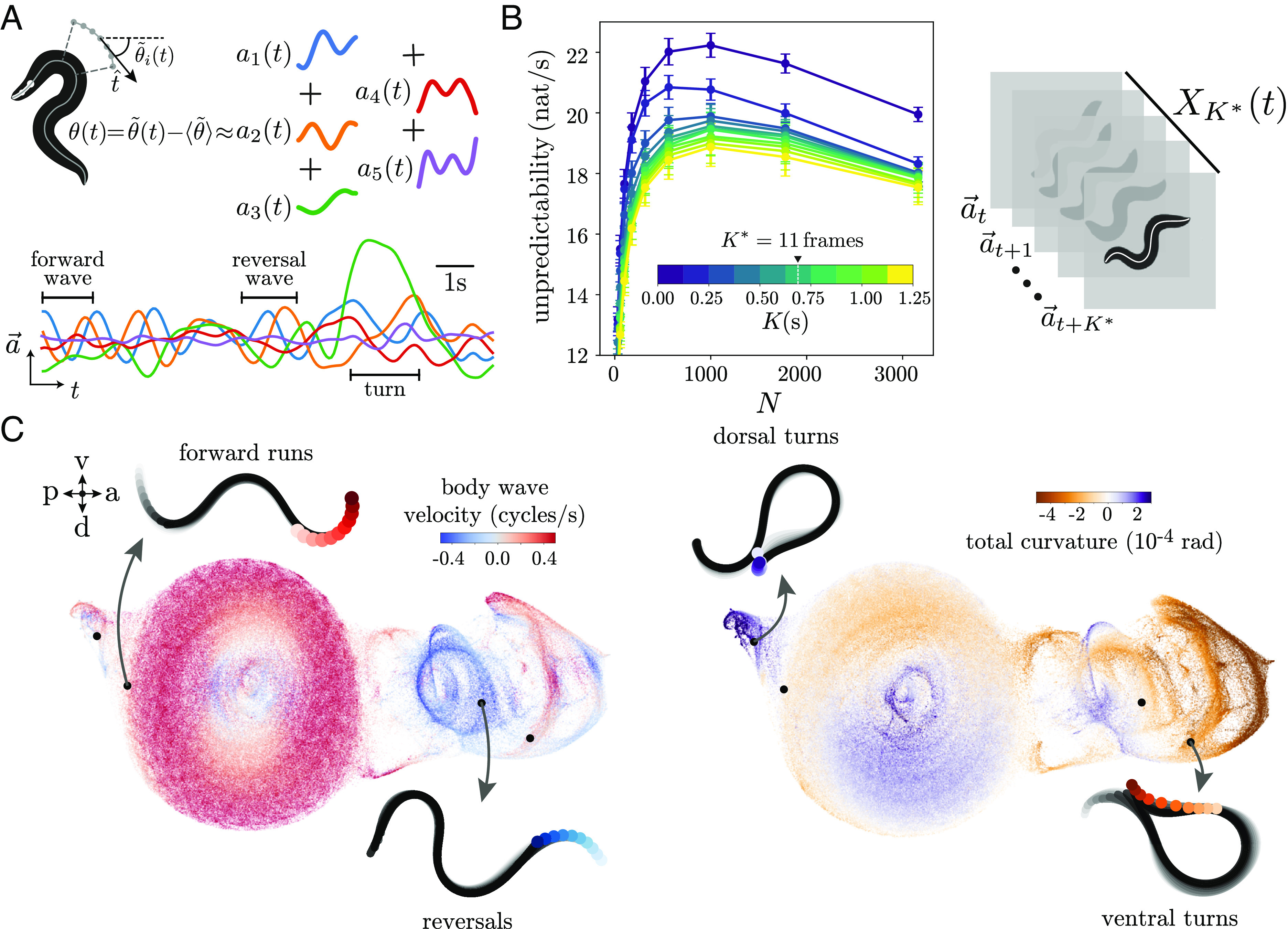
Maximally predictive posture sequences reveal the space of short-time behaviors. (A, *Left*) We represent the posture at each frame t using an “eigenworm” basis (20). We extract the worm’s centerline and measure the tangent angles between body segments ${\tilde{\theta }}_{i}\left(t\right)$. We then subtract the average angle to obtain a worm-centric representation $\theta =\tilde{\theta }-\left\langle \tilde{\theta }\right\rangle$ and project the mean-subtracted angles onto a set of eigenworms (20), obtaining a 5D (eigenworm coefficient) time series $\vec{a}\left(t\right)$. (*A*, *Bottom*) A segment of the time series: in food-free conditions, the short time scale behavior roughly consists of forward and reversal waves (clearly visible as oscillations in $a_{1}$ and $a_{2}$), as well as sharp turns (characterized by large amplitude $a_{3}$^28^). (*B*) Entropy rate as a function of the sequence length K and number of partitions N. We partition each sequence space (indexed by length K) into N microstates using k-means clustering and compute the entropy rate of the resulting Markov chain. The curves collapse after K∼0.5s, indicating that the entropy rate is approximately constant meaning that there is no further gain in predictability by including more time delays. We choose $K^{*}=11\;\;\text{frames}=0.6875\;\;\text{s}$ to define a maximally predictive sequence space $X_{K^{*}}$. Error bars are bootstrapped SDs across worms. (*C*) We visualize $X_{K^{*}}$ by projecting onto two-dimensions using UMAP (31), and coloring each point by the body wave phase velocity $\omega =-\frac{1}{2\pi }\frac{d}{\mathrm{dt}}\left[\;{\text{tan}}^{-1}\left(a_{2}/a_{1}\right)\right]$ (20) (*Left*) and the overall body curvature (*Right*) obtained by summing the tangent angles $\theta_{i}$ along the body $\gamma =\sum_{i}\theta_{i}$. Each point on this space corresponds to a $K^{*}$ sequence of postures (from light to dark colors), and different short-time behaviors naturally correspond to different regions on the projection.

We employ a previously analyzed dataset (28) composed of 35 min recordings of 12 young-adult lab-strain N2 worms freely moving on an agar plate, sampled at δt=1/16s (a total of 33,600 frames per worm). From high-resolution videos, we measure the worm’s body posture using a rich low-dimensional representation of the centerline, expressed as five “eigenworm” coefficients $\vec{a}=\left[a_{1},a_{2},a_{3},a_{4},a_{5}\right]\in R^{5}$ (20, 28), Fig. 1*A*. Posture itself already carries important behavioral information, for example, the joint distribution of the first two eigenworm modes is approximately circular, describing the phase of the crawling locomotory wave (20). But posture dynamics carries even more information, such as the direction of the wave which distinguishes forward crawls from reversals.

We capture posture dynamics through a maximally predictive sequence space (6) constructed by stacking K delays of the posture time series, and increasing K until we have maximized the predictability of the resulting dynamics, as measured by the entropy rate, Fig. 1*B*, *Left*. While other quantitative approaches to behavior also start with short dynamical motifs (29, 30), our work is distinguished by a focus on maximal predictability to determine the motif length. To estimate the entropy rate, we partition each sequence space (indexed by K) into N microstates $s_{i}$ using k-means clustering so that the posture dynamics now appear as transitions between microstates, a Markov chain. We build the partition using all 12×33,600 observations so that the microstate labeling is consistent across worms. We approximate the entropy rate as that of the Markov chain inferred from each worm’s symbolic sequence and choose the largest N before finite size sampling reduces the estimated entropy.^*^ This “maximum entropy” partitioning (with $N^{*}=1,000$ partitions) requires a large number of microstates, but enables our model to be maximally expressive within the limits of the data (which set the onset of finite size effects in the entropy estimation). After K≈8frames=0.5s and the entropy rate curves start to collapse, and we set $K^{*}=11\;\;\text{frames}=0.6875\;\;\text{s}$ to define the maximally predictive sequence space $X_{K^{*}}$ of dimensions 5×11, Fig. 1*B*, *Right*; lengthening the sequences does not increase predictability, *SI Appendix*, Fig. S1*A*. While *SI Appendix*, Fig. S1*A* suggests that $K^{*}=6\;\;\text{frames}$ is enough to maximize predictability, we choose a slightly higher value of $K^{*}$ to ensure that we fully resolve the predictive information lying in past posture measurements.

We visualize the high-dimensional $X_{K^{*}}$ by projecting into two dimensions using the Uniform Manifold Approximation and Projection (UMAP) manifold learning algorithm (31) (*SI Appendix*, *Materials and Methods*), Fig. 1*C*. Color-coding according to the worm’s body wave phase velocity $\omega =-\frac{1}{2\pi }\frac{d}{\mathrm{dt}}\left[\;{\text{tan}}^{-1}\left(a_{2}/a_{1}\right)\right]$, (20) Fig. 1 C, *Left*, and overall curvature (obtained by summing the tangent angles along the body), $\gamma =\sum_{i}\theta_{i}$, reveals that distinct short-time behavioral motifs, corresponding to forward, reversal, and turning movements, naturally correspond to different regions of the maximally predictive state space, Fig. 1*C*, *Right*). In other words, while the instantaneous posture itself $\vec{a}\left(t\right)$ is not enough to disentangle different behaviors, a point in the sequence space $X_{K^{*}}\left(t\right)$ uniquely corresponds to a particular short-time behavioral motif.

## The Markov Worm

Our combination of sequence embedding and partitioning naturally results in a symbolic dynamics where each state probabilistically transitions to a new state (6). These dynamics are described through the master equation

[1] $$\begin{array}{c} p_{j}\left(t+\tau \right)=\sum_{i}P_{\mathrm{ij}}\left(\tau \right)\;p_{i}\left(t\right), \end{array}$$

where $p_{i}\left(t\right)$ is the probability of observing state $s_{i}$ at time t, and $P_{\mathrm{ij}}\left(\tau \right)$ is a transition matrix constructed by counting transitions between microstates $s_{i}\left(t\right)$ and $s_{j}\left(t+\tau \right)$ after a delay τ. We note that P is a stochastic matrix so that each transition probability $P_{\mathrm{ij}}\geq 0$ and $\sum_{j}P_{\mathrm{ij}}=1$ for all microstates i. The waiting time τ is a free parameter which can be as short as δt, as we used previously for the maximally predictive embedding, and we will leverage this freedom to emphasize long-lived aspects of the worm’s movement. We have thus transformed the behavioral dynamics into a (generally nonequilibrium) Markov chain with many states, and where each state itself carries short-time dynamical information.

**Fig. 2.**
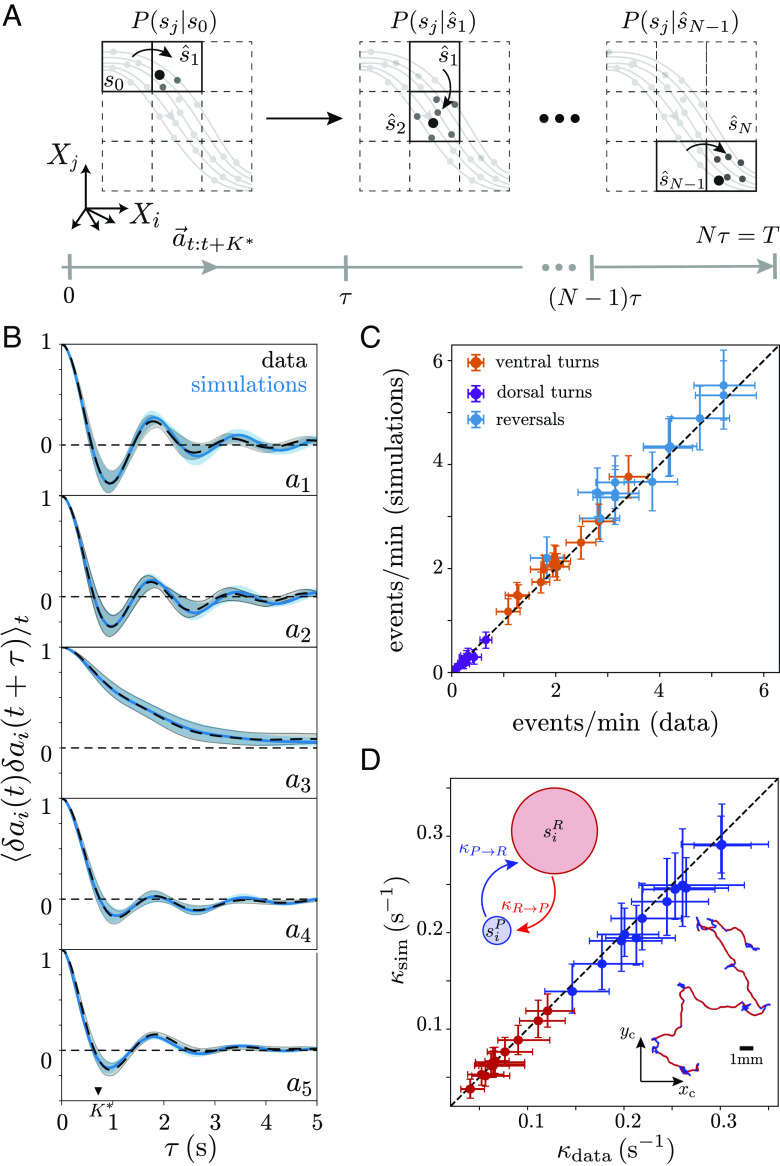
A Markov model captures posture dynamics across timescales. (*A*) Schematic of the simulation method. Starting from the initial microstate of each worm $s_{0}$, the next microstate is obtained by sampling from $P_{0j}\left(\tau^{*}\right)$. We add new microstates in the same fashion, resulting in a symbolic sequence of length $N=T\delta t/\tau^{*}$. Within each microstate, we randomly choose a point in the maximally predictive sequence space, $X_{K^{*}}$ and use this point to identify the associated sequence of body postures ${\vec{a}}_{t:t+K^{*}}$. (*B*) The simulated posture dynamics accurately predicts the autocorrelation function of the “eigenworm” coefficient time series. Shaded regions correspond to 95% CIs of the estimate of the mean autocorrelation function bootstrapped across worms. (*C*) The simulated posture dynamics accurately predicts the average rate of behavioral events across worms. We estimate the number of reversals, ventral turns, and dorsal turns per unit time and compare the result obtained from the data with simulations of each of the 12 recorded worms, finding excellent agreement. Error bars correspond to bootstrapped 95% CIs on the estimates of the mean. (*D*) On longer time scales, worms transition between relatively straight “runs” and abrupt reorientations through “pirouettes” (32), which are combinations of reversals and turns (*Inset*, *BottomRight*). We assign each microstate to either a “run” or a “pirouette” by leveraging the inferred transition matrix to identify long-lived stereotyped sequences of posture movements (*Coarse-Graining Behavior through Ensemble Dynamics*). We then estimate the mean transition rates from the run to the pirouette state $\kappa_{R\to P}$ (red) and from the pirouette to the run state $\kappa_{P\to R}$ (blue) for each of the 12 recorded worms. Data error bars are 95% CIs of the mean bootstrapped across run and pirouette segments, simulation error bars are 95% CIs of the mean bootstrapped across 1,000 simulations.

As described in the previous section, for the worm’s posture dynamics, we set the number of microstates as $N^{*}=1,000$ so as to maximize the amount of information with respect to the partitioning, Fig. 1*B*. We then set the transition time as $\tau^{*}=0.75\;\;\text{s}$ so that the relaxation times of the long-lived dynamics are approximately independent of τ, as rigorously true in a Markov process (*SI Appendix*, Fig. S1 *B* and *C* and *Materials and Methods*). In practice, from the $\text{33,600}-K^{*}-\tau^{*}$ observations of each worm, we count how many times state $s_{i}$ reaches state $s_{j}$ in a timescale $\tau^{*}$. Since $\tau^{*}$ is much shorter than the mixing time of the dynamics, most transitions occur locally in $X_{K^{*}}$, so we expect that the number of nonzero transition probabilities is much smaller than $N^{*}\times N^{*}$. Indeed we find that from each $s_{i}$, the dynamics only reaches ∼10 other states within $\tau^{*}$ yielding transition matrices with ≈10,000 entries, *SI Appendix*, Fig. S4. Despite the conceptual simplicity of our Markov chain model, Eq. **1**, we next show that it accurately predicts *C. elegans* foraging behavior across scales, from fine-scale posture movements to long time scale transitions between behavioral states, Fig. 2 and *SI Appendix*, Fig. S4.

### Predicting Behavior Across Scales.

Starting from the initial state of an individual worm, which corresponds to a discrete microstate $s_{i}\left(t=0\right)$ of the $K^{*}$ space of posture sequences, we simulate symbolic sequences by sampling from the conditional probability distribution $P^{w}\left(s_{j}|{\hat{s}}_{i}\right)$, Fig. 2*A*, where ${\hat{s}}_{i}$ is the current microstate, $s_{j}$ are all possible future microstates after a time scale $\tau^{*}$ and $P^{w}\left(s_{j}|{\hat{s}}_{i}\right)$ is the i-th row of the transition matrix inferred for worm w. The result is a sequence of microstates with the same duration as the worm trajectories, but with a sampling time $\delta t=\tau^{*}$. From each microstate, we can then obtain a nearly continuous time series of “eigenworm” coefficients $\left\{\vec{a}\left(t\right)\right\}$ through the sequence of $K^{*}$ postures in each state $X_{K^{*}}$ (note that $K^{*}$ and $\tau^{*}$ are quite close in this case). These dynamics are effectively diffusive in the space of posture sequences: hopping between microstates according to the Markov dynamics, followed by random selection from the set of posture sequences $X_{K^{*}}^{i}$ within each visited microstate $s_{i}$. The posture time series generated through this procedure are nearly indistinguishable from the data, *SI Appendix*, Fig. S2 and Movie S1. Quantitatively, the autocorrelation functions of the simulated time series, Fig. 2*B* and *SI Appendix*, Fig. S4*A*, capture the correlations observed in the data, and the distribution of mode coefficients agrees with the steady-state distribution, *SI Appendix*, Fig. S3.

In addition to the finescale posture dynamics, our model also predicts the rate at which forward movements are interrupted by biologically relevant behaviors (33) such as reversals, dorsal turns, or ventral turns (identified by thresholding the body wave phase velocity (20) and the overall body curvature, see *SI Appendix*, *Materials and Methods*), Fig. 2*C* and *SI Appendix*, Fig. S4*B*.

At larger spatiotemporal scales, the foraging random-walk can be coarsely split into forward “runs” interrupted by “pirouettes,” a single or sequence of sharp centroid turns, which are used by the worm to reorient itself between longer-lived runs (32). We note that the term “pirouette” was originally introduced to broadly describe the reversals, deep turns, and combinations of reversals and deep turns seen during chemotaxis (32). This definition has also been applied to the worm’s navigation behavior in food-free environments (34), similar conditions to the recordings described here. Here, we identify long-lived behavioral states from posture dynamics by using the inferred transition matrix to find sets of movements that typically occur in a sequence (we call these “macrostates,” see the following section *Coarse-Graining Behavior through Ensemble Dynamics*). At the two-state level, the macrostates split the centroid trajectory into relatively straight paths and more abrupt reorientations, and we thus identify these macrostates as the posture-scale expression of “runs” and “pirouettes” described above, Fig. 2*D*, *Inset*. We estimate the kinetic transition rates from runs-to-pirouettes $\kappa_{R\to P}$ and from pirouettes-to-runs $\kappa_{P\to R}$ and find close agreement between data and simulations across worms, Fig. 2*D* and *SI Appendix*, Fig. S4*C*.

### Posture to Path.

Our Markov model on posture sequences is remarkably powerful at predicting worm behavior from an egocentric point of view. However, to understand how such dynamics relate to biological function, we need to connect posture dynamics to motility in the 2D space. With such a bridge we could connect the neuromechanical control of posture with movement strategies such as optimal search. Adding posture-to-path to our predictive Markov models offers the possibility of going beyond the limits of experimental observations and offering accurate in silico trajectories for studying the navigational role of distinct coarse-grained behaviors. To do so, however, we must first connect posture deformations with movement in the environment.

Following previous work (35), we approximate the interaction between the worm’s body and the viscous agar surface through resistive force theory (RFT) (36). This phenomenological approach assumes that each segment along the body experiences independent drag forces. Despite its simplicity, this approximation has been successfully applied to predict the motility of various organisms in viscous fluids (37–39) and granular materials (40).

**Fig. 3.**
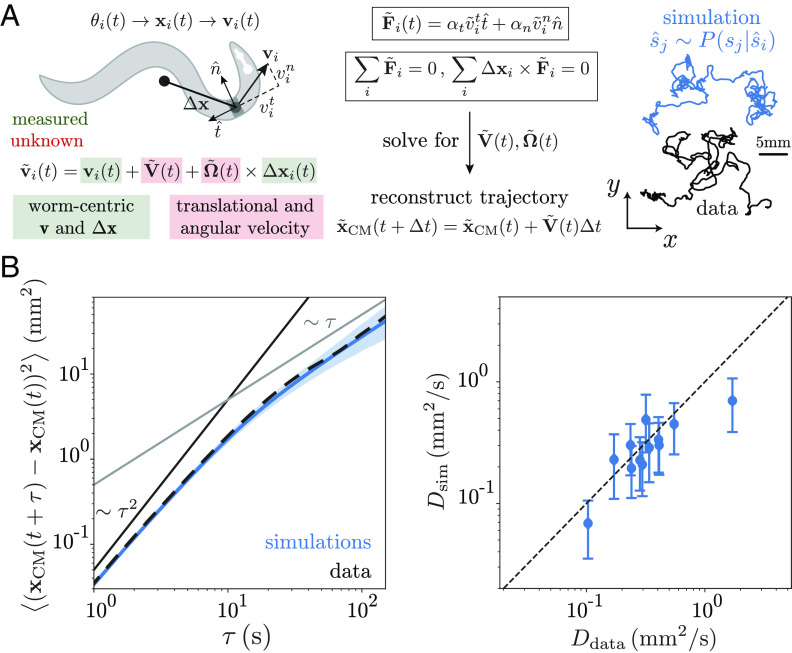
Recovering foraging trajectories from posture simulations. (*A*) We use RFT (35) to translate the simulated posture dynamics into movement. We simulate the Markov chain posture dynamics as described in Fig. 2. At each frame t of the simulated posture time series, we reconstruct the coordinates of the i-th segment of the skeleton, $x_{i}\left(t\right)$, from the tangent angles $\theta_{i}\left(t\right)$, which are themselves a linear combination of eigenworms with the mode weights particular for each frame. The measured velocities, $v_{i}$, in the frame-of-reference of the worm, correspond to subtracting the overall velocity $\tilde{V}\left(t\right)$ and angular velocity $\tilde{\Omega }\left(t\right)$ of the worm from the lab-frame velocities ${\tilde{v}}_{i}\left(t\right)$, which are unknown. Following the results ref. 35, we recover the lab-frame translational $\tilde{V}\left(t\right)$ and angular $\tilde{\Omega }\left(t\right)$ velocities by leveraging resistive force theory to equate the tangent and normal forces acting in each local segment to the local velocities and imposing a zero net-force and net-torque condition. The ratio between tangent $\alpha_{t}$ and normal $\alpha_{n}$ damping coefficients is the single free parameter $\alpha =\alpha_{t}/\alpha_{n}$, which we find by minimizing the distance between simulated and real trajectories, *SI Appendix*, Fig. S5*A*. We show an example worm trajectory (black), as well as simulated trajectories reconstructed from posture time series generated from the operator dynamics (blue). (*B, Left*) Mean square displacement of centroid trajectories obtained from model simulations (blue) and data (black) for an example worm. Simulation error bars are 95% CIs of the mean bootstrapped across 1,000 simulations. The results for all 12 worms analyzed here can be found in *SI Appendix*, Fig. S5*B*. (*B, Right*) Effective diffusion coefficients obtained from simulations and from the data. We estimate D by fitting the slope of the mean square displacement curves in the range τ∈[60,100]s, MSD(t)=4Dt. Errors are SDs of the estimated diffusion coefficients across simulations.

To propel the worm, we first reconstruct the skeleton positions in each frame $x_{i}\left(t\right)$ from the instantaneous tangent angles $\theta_{i}\left(t\right)$ along each body segment i, Fig. 3*A*, *Left*. From these, we derive the worm-centric velocities $v_{i}\left(t\right)=x_{i}\left(t+1\right)-x_{i}\left(t\right)$ and displacements with respect to the center-of-mass position $x_{\;\text{CM}}$, $\Delta x_{i}\left(t\right)=x_{i}\left(t\right)-x_{\;\text{CM}}\left(t\right)$. This results in an expression for the underlying velocities at each body segment as a function of the measured worm-centric v and Δx and unknown overall translational $\tilde{V}\left(t\right)$ and angular $\tilde{\Omega }\left(t\right)$ velocities. As in ref. 35, we use linear resistive force theory to decompose the force acting on each body segment into tangent and normal components ${\tilde{F}}_{i}\left(t\right)=\alpha_{t}{\tilde{v}}_{i}^{t}\hat{t}+\alpha_{n}{\tilde{v}}_{i}^{n}\hat{n}$, with drag coefficients $\alpha_{n}$ and $\alpha_{n}$, Fig. 3 *A*, *Middle*. Using this approximation, we can then recover the unknown underlying velocities ${\tilde{v}}_{i}\left(t\right)$ by imposing a zero net force and torque condition. The only free parameter in this model is the ratio between the normal and tangential drag coefficients $\alpha =\alpha_{n}/\alpha_{t}$, which we infer by minimizing the distance between the reconstructed centroid trajectories and the real data (*SI Appendix*, *Materials and Methods*), *SI Appendix*, Fig. S5*A*. In agreement with the results of Keaveny et al. (35), we find that in such food-free conditions, the value of α that optimizes the reconstruction of centroid trajectories is $\alpha^{*}=30\;\left(29,31\right)$. Using such $\alpha^{*}$, we then reconstruct the centroid path corresponding to the posture time series simulated according to our Markov chain and show that these qualitatively resemble real worm trajectories, Fig. 3*A*, *Right*.

To further quantify the similarity between the centroid trajectories reconstructed from posture simulations and the data, we estimate the mean squared displacement $\;\text{MSD}\left(\tau \right)=\langle {\left(x_{\;\text{CM}}\left(t+\tau \right)-x_{\;\text{CM}}\left(t\right)\right)}^{2}\rangle$, which exhibits a transition between superdiffusive (nearly ballistic) and diffusive behavior between 10 s and 100 s (41–44), Fig. 3*B*, *Left* and *SI Appendix*, Fig S5*B*. The foraging trajectories corresponding to the operator-based simulations accurately capture the MSD across a wide range of scales, including the ballistic-to-diffusive transition. To further assess the quality of the simulations, we estimate an effective diffusion coefficient by fitting MSD=4Dτ in the linear regime^†^ and find that, across worms, the resulting diffusion coefficients obtained from simulations closely follow the data, Fig. 3*B*, *Right*. Our results demonstrate that it is possible to predict long time scale diffusive properties from fast posture dynamics in animals.

## Coarse-Graining Behavior through Ensemble Dynamics

As highlighted in Fig. 2, *C. elegans* foraging behavior exhibits multiple time scales: from the body waves that define short-time behaviors (e.g., forward, reversal, turns) to longer-time sequences (e.g., run, pirouette) that the worm uses to navigate its environment. Typically, these longer-time sequences have been identified phenomenologically by setting thresholds on heuristically defined quantities, as was done in Fig. 2*C*. Here, we show that it is possible to reveal the multiple scales of *C. elegans* locomotion directly from the posture dynamics. Intuitively, stereotyped behaviors correspond to regions of the behavioral space that the animal visits often. In an analogy with statistical mechanics, we can imagine behavior as evolving on a complex potential landscape, where each well corresponds to a particular stereotyped behavior, and the barrier heights set the transition time scales. Such a picture emerges naturally when analyzing the dynamics through an ensemble approach, and we leverage our inferred Markov chain to directly identify metastable behaviors.

### “Run-and-Pirouette”.

The eigenvalues of the transition matrix provide direct access to the long time-scale properties of the dynamics, even when these are not directly apparent from the original trajectories or the equations of motion. The real part of the eigenvalues $\left\{\lambda_{i}\right\}$ of $P_{\mathrm{ij}}\left(\tau^{*}\right)$ quantify the relaxation time to the steady state,

[2] $$\begin{array}{c} \Lambda_{i}^{-1}=\frac{-\tau^{*}}{\log\;\text{Re}(\lambda_{i})}. \end{array}$$

**Fig. 4.**
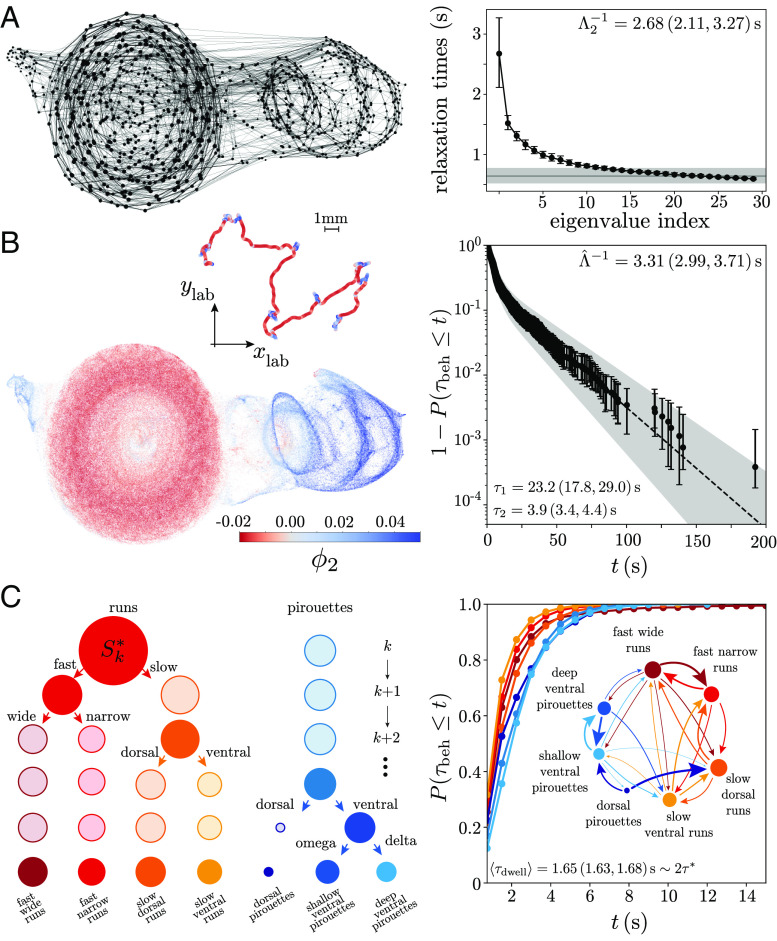
Slow modes for coarse-graining: from “runs-and-pirouettes” to stereotyped body waves. (*A, Left*) Schematic of the inferred Markov chain. We partition the maximally predictive sequence space $X_{K^{*}}$ (11×5 dimensions) into Voronoi cells, here represented as points in the 2-dimensional UMAP embedding space. The size of each point is proportional to the probability of visiting a given microstate $s_{i}$, and the line width corresponds to the probability of transitioning among distinct states after τ=1frame (we only show transitions with $P_{\mathrm{ij}}>0.025$ for simplicity). (*A, Right*) Relaxation timescales obtained from the 30 eigenvalues with the largest real part of $P_{\mathrm{ij}}\left(\tau^{*}\right)$ with $\tau^{*}=0.75\;\;\text{s}$. The horizontal gray bar is the largest nontrivial eigenvalue of the transition matrix computed from a shuffled symbolic sequence (see *SI Appendix*, *Materials and Methods* for details). Error bars are 95% CIs bootstrapped across worms. (*B, Left*) We color the sequence space and an exemplar centroid trajectory (*Inset*) by the first nontrivial eigenvector of the reversibilized transition matrix $\phi_{2}$, which optimally separates metastable states (6, 46). Positive values correspond to combinations of reversals and turns that reorient the worm, these are “pirouettes,” while negative values correspond to “forward runs.” (*B, Right*) We use $\phi_{2}$ to identify two coarse-grained regions, which we denote as “macroscopic” behavioral states and find that the complementary cumulative distribution function of the resulting dwell times $1-P(\tau_{\;\text{dwell}}\leq t)$ is characterized by two time scales, which we extract by fitting a sum of exponential functions. The inferred time scales are in agreement with previous phenomenological observations of worm behavior (32) and result in a relaxation time ${\hat{\Lambda }}^{-1}={\left(\tau_{1}^{-1}+\tau_{2}^{-1}\right)}^{-1}=3.31\;\left(2.99,3.71\right)\;\;\text{s}$ (47) that agrees with the largest eigenvalue of $P_{\mathrm{ij}}\left(\tau^{*}\right)$, $\Lambda_{2}^{-1}$, within error bars. The timescale errors and error bars are 95% CIs bootstrapped across events. (*C*) The subdivision process. At each iteration step, we subdivide the metastable state with the largest measure, $S_{k}^{*}$, along the first nontrivial eigenvector obtained from the transition probability matrix conditioned only on the states within that metastable state (48). This results in a top–down subdivision of behavior that follows the structure of an effective free energy landscape. The size of the circles represents the relative measure in each state. For interpretability, we stop at the fifth subdivision, yielding 7 “mesoscopic” states (characterized below). (*C, Right*) Cumulative distribution of the mesoscopic state dwell times: The duration is short $\left(\approx 2\tau^{*}\right)$. The *Inset* shows a transition diagram in which the size of the nodes and the edge widths are proportional to the measure in each behavior and the transition probabilities, respectively (transition probabilities <0.05 are not graphed for simplicity).

The spectrum of relaxation times is shown in Fig. 4*A*, *Right* and exhibits both an isolated, longer-lived mode with $\Lambda_{2}^{-1}=2.68\;\left(2.11,3.27\right)\;\;\text{s}$, as well as an additional ≈10 significant modes.

The eigenvectors corresponding to the long-lived dynamics provide continuous reaction coordinates for a natural coarse-graining. In Fig. 4*B* we color-code the maximally predictive sequence space according to $\phi_{2}$; comparing with Fig. 1*C*, we find that negative values of $\phi_{2}$ (red) correspond to positive phase velocities and low curvatures, indicative of “forward runs,” while positive values of $\phi_{2}$ (blue) generally correspond to sequences of reversals, dorsal and ventral turns used during abrupt reorientations, see *Inset* of Fig. 4*B*. The *Inset* shows an example 10-min-long centroid trajectory color coded by the projection along $\phi_{2}$. Negative projections occur during “runs,” while positive values are found during abrupt reorientation events composed of sequences of reversals and turns. We thus obtain a slow reaction coordinate that captures the dynamics along a posture-based expression of a “run-and-pirouette” axis.

The remaining eigenfunctions reveal substructures within “runs” and “pirouettes.” For example, in *SI Appendix*, Fig. S6, we show the maximally predictive sequence space color coded by the following 3 long-lived eigenvectors, $\left\{\phi_{3},\phi_{4},\phi_{5}\right\}$. We see that $\phi_{3}$ differentiates dorsal and ventral turns, $\phi_{4}$ differentiates turning and reversals, and $\phi_{5}$ differentiates the compound motifs of shallow turns following a pause, from reversals that are followed by deep δ-turns. Projections onto the slow eigenvectors thus reveal continuous modulations along the short nematode locomotory movements first observed by Croll (19), *SI Appendix*, Fig. S6, as well as longer-lived, compound motifs, such as runs-and-pirouettes, Fig. 4*B*.

While the continuous variation along these slow modes neatly highlights the temporal organization of distinct features of worm behavior, we can also use them to identify stereotyped behavioral states as “macroscopic” metastable sets (49, 50): groups of microstates that transition more often within rather than between groups. Stereotyped behaviors correspond to finescale movements that occur more often than not in a sequence. In other words, stereotypy implies a timescale separation between variations on what we call a behavioral state, and transitions among distinct stereotyped behaviors. This notion of stereotypy is naturally captured by the notion of coherent/metastable sets, which can be directly identified through the eigenvalues and eigenvectors of the Markov chain. In this way, the structure of these sets and the kinetics between them offer a coarse-graining of behavior that directly follows the multiple timescales of the dynamics. We note that this construction provides a precise meaning to the discreteness of such behavioral states: the discrete approximation is appropriate at a particular timescale of interest. Faster timescales require finer scale states, while longer timescales can be neatly approximated by larger coarse-grained states.

More principledly, we start by identifying long-lived behavioral states as almost invariant sets (46) in the reconstructed state space. These sets are optimally determined by the second eigenvector $\phi_{2}$ of the time-symmetric reversibilized transition matrix $P_{r}$, which we infer from the ensemble of worms (*SI Appendix*, *Materials and Methods*) (51, 52). We search along $\phi_{2}$ for a single threshold that maximizes the metastability of both resulting coarse-grained sets (*SI Appendix*, *Materials and Methods*) (6), *SI Appendix*, Fig. S7, and we identify the resulting two macrostates as “run” and “pirouette.” The complementary cumulative distribution of the time spent in either of the two states $1-P(t_{\;\text{beh}}\leq t)$ is roughly characterized by two time scales, Fig. 4*B*, *Right*, fit by a sum of exponential functions and in excellent agreement with previous phenomenological observations (32). In addition, these transition timescales are related to the timescale of relaxation to the steady-state distribution as ${\hat{\Lambda }}^{-1}=1/\left(\tau_{1}^{-1}+\tau_{2}^{-1}\right)=3.313\;\left(2.985,3.709\right)\;\;\text{s}$ (47), which agrees with the relaxation times of the transition matrix within statistical accuracy, Fig. 4*A*, *Right*. In our analysis “run-and-piroutte” kinetics emerge directly from worm-centric posture dynamics, without any positional information.

### “Run(s)-and-Pirouette(s)”.

While dividing the dynamics along $\phi_{2}$ identifies the longest lived states, the existence of other significant eigenvectors, Fig. 4*A*, indicates that there are finer-scale divisions. To identify such states in a way that naturally reflects the behavioral landscape, we iteratively subdivide the largest metastable state into faster mesoscopic states (48), Fig. 4*C*. In practice, we identify the most-visited metastable state, construct a reversibilized transition matrix using only the microstates within that metastable set, and use its first nontrivial eigenvector to subdivide the dynamics (see *SI Appendix*, *Materials and Methods* for details). This is akin to subdividing a free-energy landscape; at each iteration, we subdivide the system along the largest energy barrier within the highest measure basin. We also note that our subdivisioning proceeds from the longest-lived states down rather than from the shortest-lived states up, where the latter is more common in behavior coarse-graining approaches (29, 53, 54).

In the foraging behavior of *C. elegans*, beyond the initial division into runs and pirouettes (which we denote as “macroscopic” states), we further subdivide the dynamics into 7 “mesoscopic” interpretable states: Four distinct run states and three subdivisions of the pirouette state Fig. 4*C* and *SI Appendix*, Fig. S8. The run state essentially splits into two fast states and two slower states, which can be distinguished either by the wave length of the body, or by having a particular bias toward the dorsal or ventral sides: the dorsally biased slow state is akin to a head-casting state (55), while the ventrally biased stated is akin to a “dwelling” state (56–58), with incoherent head and tail movements and no propagating wave (13). On the other hand, the pirouette state neatly splits into dorsal turns, deep ventral δ-turns, and reversals followed by shallow Ω-turns.

These mesoscopic states that decorate the worm’s foraging behavioral landscape are short-lived, with a characteristic time scale of $\left\langle \tau_{\;\text{dwell}}\right\rangle =1.65\;\left(1.63,1.68\right)\;\;\text{s}\approx 2\tau^{*}$, Fig. 4*C*, *Right*. The transition diagram between them, Fig. 4*C*, *Right* and *Inset*, reveals the fine-scale organization of the worms’ foraging strategy. Further subdivisions result in even shorter-lived states, which are increasingly challenging to interpret.

## Exploring the Role of the Mesoscopic States

In the data analyzed here, worms were grown in a food-rich environment, but then placed on food-free agar plates and allowed to move without restrictions. Under these conditions, the worm’s behavior has been qualitatively described as foraging (59, 60). We apply our approach to better understand the role of the mesoscopic states in the worm’s search for food.

**Fig. 5.**
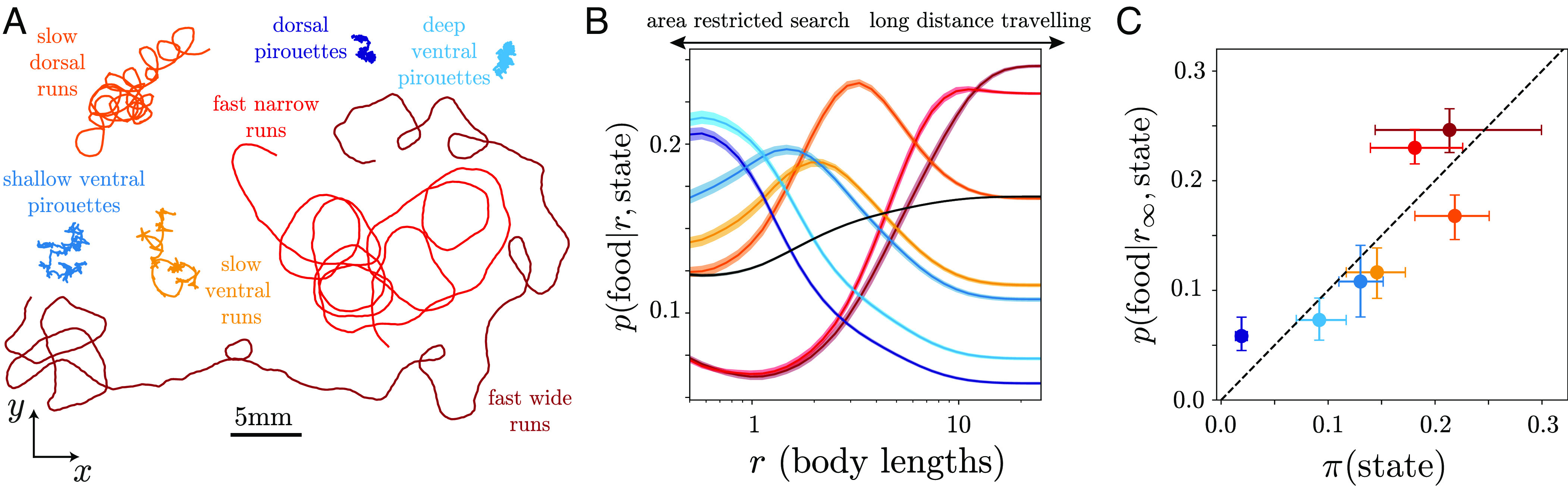
Leveraging our multiscale description to explore the role of behavioral states. We simulate long trajectories while forcing the worm’s dynamics to remain within a single mesoscopic state S obtained through a subdivision of the behavioral space into 7 states, Fig. 4*C*. We proceed as in Figs. 2 and 3, but sample each new state according to a transition matrix built only within the partitions belonging to S, ${\hat{s}}_{j}\left(t+\tau \right)∼P_{S}\left(s_{j}|{\hat{s}}_{i}\left(t\right)\right),\;i,j\in S$. (*A*) Example 10-min-long centroid trajectories of each state. (*B*) Probability of finding food in a uniform food patch of radius r for a particular state. We find that the most efficient behavioral states change depending on the radius of the food distribution: at short distances, “pirouette” and slow “run” states provide better chances of finding food, whereas at larger distances, the two fast “run” states are most efficient. The black line represents the strategy of an average worm $p\left(\;\text{food}|r,\;\text{average worm}\right)=\sum_{S}\pi \left(S\right)p\left(\;\text{food}|r,S\right)$, obtained as a weighted average over the probability of finding a worm in a particular mesoscopic state S, π(S). While the worm’s movement strategy is more likely to find food sources scattered at larger distances, the ensemble of mescoscopic states can be utilized for foraging success across scales. (*C*) The likelihood of finding food in a large uniform food patch for each of the states closely matches the probability of finding a worm in a particular state.

We use the Markov model to simulate in silico worms that are forced to remain in a particular mesoscopic state and the posture-to-path framework to investigate the properties of the trajectories resulting from the posture dynamics in each of the states. We can simulate trajectories that are much longer than those observed in the data, Fig. 4*C*, *Right*, allowing us to dissect how different states produce distinct large-scale tracks. In Fig. 5*A*, we show simulated 10min long trajectories for each of the 7 mesoscopic states. Notably, the difference in posture wavelengths exhibited by the two fast “run” states, *SI Appendix*, Fig. S8*B*, results in dramatically different trajectories, with the longer wavelength state (fast wide runs) resulting in overall straighter paths and the shorter wavelength state (fast narrow runs) resulting in ventrally biased curved trajectories with a diameter that is several times the body length and a period orders of magnitude longer than the body wave period, *SI Appendix*, Fig. S9. Interestingly, the dorsally biased slow state also results in loopy trajectories, but with a shorter diameter and faster recurrence time. In addition, the ventrally biased “dwelling”-like slow state (56–58) with its frequent head retractions results in a denser sampling of a local patch. Finally, the three “pirouette” states result in a denser sampling of space and a reduced centroid displacement.

We next interrogate the efficiency of each of the 7 mesoscopic states at encountering food uniformly distributed within a disc of radius r around the initial position, Fig. 5*B*, a simple but informative condition. We find that the “pirouette” states as well as the slow “run” states are most efficient at finding food at shorter distances, while at larger distances, the two fast “run” states perform best. Such a differential use of behaviors, evocative of an exploration–exploitation trade-off, is also seen in nature. Upon encountering food, *C. elegans*, as well as many other species, engage in area restricted search, which is characterized by shorter paths and a high frequency of large angle turns (34, 43, 59, 61–65). Conversely, upon removal from food, *C. elegans* lowers its turning rate (42, 66) to engage in global search or long distance travel (34, 59, 63–65).

Remarkably, we find that instead of only using the most efficient behavioral state (“fast wide runs”), worms engage in a strategy that employs each mesoscopic state in a proportion that closely matches the relative efficiency of the different states at finding food uniformly distributed in a large patch (several body lengths), Fig. 5*C*. This “probability matching” behavior has been observed across several species, including humans (see, e.g., refs. 67, 68, 69, 70, 71, 72), and emerges naturally in “multiarmed bandit” situations in which agents must decide among different actions that yield variable amounts of reward without knowing a priori the relative reward of each action (see, e.g., ref. 73).

## Discussion

We combine maximally predictive short posture sequences with a Markov chain model to bridge disparate scales in the foraging dynamics of the nematode worm *C. elegans*. Rather than seeking low-dimensional descriptions of the data directly (e.g. refs. 43, 58, and 74, 75, 76, 77), we instead first expand in representation complexity: enlarging the variable of interest to include time in the form of posture sequences and constructing a maximum entropy partition to capture as much predictive information as possible. This expansion both in time and number of microstates is similar in spirit to that currently found in large language models, though our conceptual approach is dramatically simpler.

The maximally predictive sequence space combines worm postures from roughly a quarter of the duration of a typical body wave, in agreement with previous work (22). On longer timescales, the posture-based “run-and-pirouette” navigation strategy (32, 78) derived from the inferred Markov dynamics provide an accurate and principled coarse graining of foraging behavior, disentangling motions that are confounded by centroid-derived measurements (see, e.g., ref. 79). This is particularly evident in our subdivision of the behavioral space. For example, we identify distinct “run” gaits that exhibit comparable centroid speeds, but are clearly distinguishable by the posture dynamics. Additionally, our top–down subdivision of behavior reflects the hierarchy of timescales in *C. elegans* foraging behavior (55). Our approach systematically identifies such a control hierarchy from behavioral recordings alone, connecting posture timescales to “run-and-pirouette” kinetics. It will be interesting to investigate how the mesoscopic states identified here are controlled by the nervous system of the worm, and recent advances in experimental techniques that permit simultaneous neural and behavioral imaging in *C. elegans* provide an exciting path toward such discoveries (80–84).

The power of our modeling approach is in its simplicity; we bridge scales using a simple but effective Markov model, and this is only possible by recognizing and exploiting the mutual dependence between modeling and representation. Instead of directly modeling the posture time series (which can require higher-order and highly non-linear terms, see e.g., ref. 20), we search for maximally predictive states such that a simpler Markovian description can nevertheless accurately predict behavior. These emergent Markov dynamics offer a promising and powerful demonstration of quantitative connections across the hierarchy of movement behavior generally exhibited by all organisms (76, 85).

By finely partitioning the space of posture sequences, we encode continuous nonlinear dynamics through a Markov chain with a large number of states. This is analogous to building a hidden Markov model (HMM), but one in which the “hidden” states are actually observable (through time delays of our observations) and for which there is a one-to-one correspondence between “hidden” states and emitted symbols: Each observation in the posture sequence $\vec{a}\left(t\right)$ uniquely determines the state $X_{K^{*}}\left(t\right)$. While HMMs are commonly used in behavioral analysis (see e.g., refs. 13, 86), they are rarely built with so many states and with the goal of correctly predicting dynamics. In particular, most approaches employ a small number of discrete behavioral states, where the number of states is a hyperparameter of the model and the discretization is not unique. In contrast, we let the data reveal the “hidden” states through time delays and set the discretization so as to maximize predictive information. In this sense, the HMM we build is unique: by revealing the hidden dynamics through time delays, the “hidden” states are uniquely determined by the observations, making the HMM unifilar (87). In other words, the “hidden” states themselves have a very definite meaning in our approach: We effectively group together “pasts” that have equal predictability over the future up to an ϵ-resolution (set by the number of partitions $N∼\epsilon^{-D_{\;\text{emb}}}$, where $D_{\;\text{emb}}$ is the intrinsic embedding dimension of the dynamics), approximating the system’s causal states (88). This set of states together with the resulting Markov chain effectively constitutes an ϵ-machine (89), the minimal maximally predictive machine. Any other HMM in which hidden states are not causal returns models that severely overestimate the complexity of the dynamics. In addition, even though we start with a large transition matrix, we can coarse-grain it by identifying which states commonly follow each other in time to generate stereotyped sequences. In this way, instead of imposing discrete states from the start (as is common with HMM approaches), we first identify a large number of predictive causal states and only then leverage the resulting Markovian dynamics to identify coarse-grained stereotyped behaviors.

Our information theoretic framework also frees us from the constraint of linearity that is commonly imposed in graphical models applied to animal behavior (such as autoregressive hidden Markov models 30). In particular, while the stereotyped states found through such models are encoded by linear dynamics, the states we identify can exhibit much more complex nonlinear dynamics, allowing us to capture longer time-scale structures in behavior.

Are Markov models enough to capture the richness of animal behavior more universally? It is important to distinguish between two sources of non-Markovianity. The first one is general and is simply induced by the fact that time series data are typically only a partial observation of the full dynamical state (6, 7). Projecting the full unobserved dynamics onto a subset of observable degrees of freedom inherently results in non-Markovian dynamics for the measured variables (2, 3, 90, 91). Such an “underembedding” might result in apparent memory when naively constructing behavioral states. The second source of non-Markovianity, which is less trivial and likely ubiquitous in behavior, derives from the fact that there may be “hidden” latent variables that modulate behavior over timescales comparable to the measurement time (5). In this case, the steady-state distribution itself is changing slowly over time, rendering the dynamics explicitly nonstationary (92).

A relevant demonstration of nonstationarity is provided by the adaptive changes in pirouette rate seen in the behavior of *C. elegans* upon removal from a food-rich environment (34, 42, 43, 59). This adaptation is present in the data we analyze and is not captured by our Markov model (92), *SI Appendix*, Fig. S10. To characterize such nonergodic latent variables requires explicitly time-dependent Markov models, which we leave for future work. We note, however, that our coarse graining can be easily extended to capture nonstationary dynamics through finding τ-dependent coherent sets that identify moving regions of the state space that remain coherent within a time scale τ (93–98).

Particularly interesting future directions include the analysis of even longer dynamics in *C. elegans* (13, 99–101), where we expect to be able to extract longer-lived behavior strategies, such as the minutes-long transitions between “roaming” and “dwelling” states in food-rich environments (56–58). Our modeling approach can also be used as means to obtain a deeper understanding of the effects of genetic, neural, or environmental perturbations on the multiple scales of *C. elegans* behavior. Indeed, the inferred transition matrices are a powerful phenotype that encapsulates multiple scales of *C. elegans* foraging behavior and has the power to reveal how behavior is affected by a given perturbation. Of particular relevance for the study of long timescales in behavior would be to focus on mutations that impair neuromodulatory pathways and are thus likely to impact the spectrum of relaxation times of the inferred Markov chain.

The effectiveness of our Markov model at capturing the nonlinear dynamics of *C. elegans* body pose, combined with the ability to translate those spatiotemporal dynamics into movement, have allowed us to investigate how different behavioral states result in distinct ways of exploring the environment at much larger scales. Our analysis recovered the two main foraging modes exhibited by *C. elegans*: one that combines different pirouette states and slow runs resulting in a local search and another one that mostly leverages fast run states to search for food more globally (34, 43, 59, 63).

We also found that the relative use of different behavioral states closely follows the relative efficiency of each state in food discovery. In fact, instead of using the behavioral state that would maximize its chances of finding food in its environment, worms match their strategy with the relative efficiency of each state. Interestingly, such a strategy, termed probability matching (71), or Thompson sampling (102), is a well-studied heuristic solution for the multiarmed bandit problem, a game in which different actions have variable rewards that are a priori unknown to the player, whose goal is to maximize total pay-out. Evidence for probability matching in decision-making tasks has been previously demonstrated in experiments in animals and humans (103, 104) and is an active area of research in cognitive science of decision-making (105).

While this strategy seems “irrational” in the context of maximizing reward in a fixed environment, that is not the condition in which worms have evolved: In ecologically relevant situations, the environment changes over time, rendering the distribution of rewards nonstationary and the subsequent sampling events correlated. Interestingly, reinforcement learning agents that have been evolved in a changing environment also develop probability matching strategies (106). In addition, it has been shown that optimal Bayesian learners engage in probability matching when they expect sampling events to have temporal dependencies (107), as is the case in most ecologically relevant scenarios in which samples of the environment are not independent, but exhibit temporal correlations (at least as a result of the actions taken by the agent). This suggests that probability matching may reflect an exploration-exploitation trade-off that robustly maximizes reward in an ever-changing environment. Our results indicate that *C. elegans* may implement such a heuristic in its foraging strategy. If the worm is indeed probability matching, it may have a way of storing its estimate of the current probability of success for each strategy (which may be reflected the dynamics itself). It will be fascinating to look for signatures of this in situations where we can experimentally adjust the pay-out probabilities. It may also be possible in a tractable organism like *C. elegans* to estimate metabolic costs to utilize each behavioral state (108). The multitude of genetic tools (109), the ability to image neurons in behaving animals (80–84), and to quantify behavior in detail makes *C. elegans* an ideal system to look inside of an organism as it experiences the world, makes decisions based on those observations and its internal model, and updates that internal model based on the outcomes of those decisions and their effect on the environment.

## Materials and Methods

Details of the reconstruction of maximally predictive posture sequences, Fig. 1, and the top–down subdivision of *C. elegans* behavior, Fig. 4, can be found in *SI Appendix*, and build upon the implementation of (6). The *SI Appendix* also includes additional information about the simulation of symbolic sequences and posture time series presented in Fig. 2. Finally, the details of the posture-to-path simulations used in Figs. 3 and 5 can also be found in *SI Appendix* and build upon the implementation of (35).

## Supplementary Material

Appendix 01 (PDF)

Movie S1. The simulated posture dynamics is virtually indistinguishable from the real worm data. We simulate a worm using the Markov model procedure of Fig. 2, and compare its posture dynamics with data starting from the same microstate. We show the curvature over time (left) for real (bottom) and simulated (top) worms, which are a priori indistinguishable from each other. We also show the corresponding RFT reconstructed skeletons (right), after subtracting the centroid position.

Movie S2. Illustration of the posture to path simulations. We simulate a worm using the Markov model procedure of Fig. 2, and translate its posture dynamics into movement using the resistive force theory approach of Fig. 3.

## Data Availability

Code for reproducing our results is publicly available: https://github.com/AntonioCCosta/markov_worm/. Data can be found in ref. 110. Analysis results data have been deposited in Kaggle (https://doi.org/10.34740/kaggle/ds/3882219). All other data are included in the manuscript and/or supporting information.

## Footnotes

^*^The entropy rate of the Markov chain should not be confused with the Kolmogorov–Sinai (KS) entropy, which is an intrinsic property of the dynamics. Indeed, the KS entropy can also be estimated using our approach, yielding accurate estimates that agree with the sum of positive Lyapunov exponents (6, 22).

^†^We note that on longer time scales the MSD exhibits the behavior of a confined random walk due to the rigid boundaries of the agar plate, which makes it nontrivial to accurately estimate the diffusion coefficient (45). We fit the diffusion coefficient in the regime τ∈[60,100], which corresponds to a time scale within which finite-size effects are negligible and the mean squared displacement is approximately a linear function of τ, MSD∼τ.
