# Supplementary material for: A Markovian dynamics for Caenorhabditis elegans behavior across scales
Source: Proc Natl Acad Sci U S A. 2024 Jul 31;121(32):e2318805121. doi: 10.1073/pnas.2318805121 (PMC11317559; doi:10.1073/pnas.2318805121)
Supplement: Supplementary file 1 — Appendix 01 (PDF) [file pnas.2318805121.sapp.pdf]

Supporting Information for

A Markovian dynamics for *C. elegans* behavior across scales

Antonio C. Costa, Tosif Ahamed, David Jordan and Greg J. Stephens

Antonio Carlos Costa
E-mail: antonioccosta.phys@gmail.com

This PDF file includes:

Supporting text
Figs. S1 to S10
Legends for Movies S1 to S2
SI References

Other supporting materials for this manuscript include the following:

Movies S1 to S2

Supporting Information Text

Materials and Methods

Software and data availability: Code for reproducing our results is publicly available: https://github.com/AntonioCCosta/markov_worm/. Data can be found in (1).

***C. elegans* foraging dataset:** We used a previously-analyzed dataset (2), in which young-adult N2-strain *C. elegans* were originally imaged at $f = 32\,\text{Hz}$ with a video tracking microscope on a food-free plate and then downsampled to $f = 16\,\text{Hz}$ to speed-up the process of resolving coiled postures (3). Worms were grown at $20°C$ under standard conditions (4). Before imaging, worms were removed from bacteria-strewn agar plates using a platinum worm pick, and rinsed from *E. coli* by letting them swim for 1 min in NGM (Nematode Growth Medium) buffer. They were then transferred to an assay plate (9 cm Petri dish) that contained a copper ring (5.1 cm inner diameter) pressed into the agar surface, preventing the worm from reaching the side of the plate. Recording started approximately 5 min after the transfer and lasted for 2100 s, for a total of $T = 33600$ frames. Each frame is converted into a 5-dimensional "eigenworm" representation $\vec{a}(t)$ by projecting the local tangent angles along the worm's centerline onto an "eigenworm" basis (2), Fig. 1(a).

Maximally predictive states: Given the measurement time series, $\vec{a}(t)$, with $t \in \{\delta t, \ldots, T\delta t\}$ and $\vec{a} \in \mathbb{R}^5$, we build a trajectory matrix by stacking K time-shifted copies of $\vec{a}$, yielding a $(T - K) \times Kd$ matrix X_K. For each K, we partition the candidate state space and estimate the entropy rate of the associated Markov chain (see below). We choose K^* such that $\partial_K h(K^*) \sim 0$, which defined X^*_K as the maximally predictive states (5), Fig. S1(a).

State space partitioning: We partition the state space constructed from the ensemble of worms into N Voronoi cells, $s_i, i \in \{1, \ldots, N\}$, through k-means clustering with a k-means++ initialization using scikit-learn (6).

Transition matrix estimation: We build a finite dimensional approximation of the Perron-Frobenius operator using an Ulam-Galerkin discretization (7). In practice, given T observations, a set of N partitions, and a transition time τ, we compute

$$C_{ij}(\tau) = \sum_{t=0}^{T-\tau} \zeta_i(X_{K^*}(t))\zeta_j(X_{K^*}(t+\tau)),$$

where $\zeta_i(x)$ are the Ulam basis functions, which are characteristic functions

$$\zeta_i(x) = \begin{cases} 1, & \text{for } x \in s_i \\ 0, & \text{otherwise} \end{cases}$$

set by the k-means clustering. The maximum likelihood estimator of the transition matrix is obtained by simply row normalizing the count matrix,

$$P_{ij}(\tau) = \frac{C_{ij}(\tau)}{\sum_j C_{ij}(\tau)},$$

which yields an approximation of the Perron-Frobenius operator.

Invariant density estimation: Given a transition matrix P, the invariant density is obtained through the left eigenvector of the non-degenerate eigenvalue 1 of P, $\pi P = \pi$: π_i is the probability of finding the system in a partition s_i.

Short-time entropy rate estimation: Given a number of partitions N and a sampling time scale $\tau = \delta t$, we estimate the Markov transition matrix P and the corresponding invariant density π as detailed above and compute the short-time entropy rate as,

$$h = -\frac{1}{\delta t}\sum_{ij} \pi_i P_{ij} \log P_{ij}. \qquad [1]$$

To obtain error bars in Fig. 1, S1(a), we estimate a transition matrix for each worm using its symbolic sequence, and then estimate its corresponding entropy rate.

Two-dimensional UMAP embedding: We use the UMAP embedding (8) as a tool to visualize the maximally predictive states of *C. elegans* posture dynamics. In a nutshell, the UMAP algorithm searches for a low dimensional representation of the data that preserves its topological structure. We use a publicly available implementation of the algorithm found in `https://github.com/lmcinnes/umap`, within which we chose the Chebyshev distance metric to compute distances in the high-dimensional space, `n_neighbors`=50 nearest neighbors and `min_dist`=0.05 as the minimum distance.

Matrix diagonalization: The high dimensionality and the sparsity of the transition matrices for large N results in numerical errors when using a naive estimator for the full spectrum of eigenvalues. In addition, since we are interested in the longest lived dynamics, we focus on finding only the n_{modes} largest magnitude real eigenvalues using the ARPACK (9) algorithm.

Choice of transition time τ^*: We choose τ^* such that the resulting Markovian dynamics approximate the long-term behavior of the system accurately, as in (5). In practice, we find the shortest transition time scale after which the inferred implied relaxation times reach a plateau, Fig. S1(b,c). For τ too short, the approximation of the operator yields a transition matrix that is nearly identity (due to the finite size of the partitions and too short transition time), which results in degenerate eigenvalues close to $\lambda \sim 1$: an artifact of the discretization and not reflective of the underlying dynamics. For τ too large, the transition probabilities become indistinguishable from noisy estimates of invariant density, which results in a single surviving eigenvalue $\lambda_1 = 1$ while the remaining eigenvalues converge to a noise floor resulting from a finite sampling of the invariant density. Between such regimes, we find a region with the largest time scale separation which also corresponds to the regime for which the longest relaxation times, Eq. (2), are robust to the choice of τ, Fig.S1(b,c). To obtain a noise floor (horizontal line in Fig. 4(a-right)), we shuffle the symbolic sequence, reestimate the transition matrix, and compute its first nontrivial eigenvalue. In the limit of infinite data, this shuffle contains only one surviving nonzero eigenvalue corresponding to the steady-state distribution (infinite relaxation time). The observation that the second largest eigenvalue is nonzero even in the shuffle is due to finite-size effects that result in small deviations from the invariant density. For further discussion see (5). To estimate error bars in Figs. 4,S1, we estimate the eigenvalues of transition matrices obtained for each worm.

Cross-validation experiments: We chose the number of partitions N^* so as to capture as much finescale detail of the dynamics without inducing finite-size effects on the estimates of the entropy rate, Fig. 1. To further attest that this choice results in generalizable models, we additionally performed cross-validation experiments as follows. We split each worm dataset into 10 segments of 3.5 minutes each, and randomly select 3 segments as a test set and the remaining 7 segments as a training set. We repeat this process over 50 random shuffles, estimating a transition matrix from the training set and making predictions over the unseen test data. In Fig. S4(a-c), we simulate the test data with the model parameters estimated in the training data, and compare such simulations against the data bootstrapping over 50 random reshuffles of train-test sets.

***C. elegans* posture simulations:** At each iteration, we sample from the conditional distribution given by the Markov chain inferred for each worm $P^w(s_j(t+\tau^*)|s_i(t))$ to generate a symbolic sequence sampled on a timescale τ^*. We then randomly sample a state space point X_{K^*} within the partition s_i, and unfold it to obtain a sequence of postures $\vec{a}_{t:t+K^*}$ at each τ^*. We can thus generate artificial posture time series with the same duration as the experimental time series (35 minutes), but with a missing frame every τ^* frames (the gap between K^* and τ^*), which we interpolate across using a cubic spline with scipy's `interpolate` package (10), and smooth with a cubic polynomial and a window size of 11 frames using the `signal.savgol_filter` package from Scipy (10). We then take the simulated $\vec{a}(t)$ time series and transform it back to the tangent angles at each body segment $\theta_i(t)$ using the "eigenworms" (2).

Estimating the rate of reversals, dorsal and ventral turn events: Reversal events where identified as segments in which the absolute value of the worms' overall curvature $\gamma(t) = \sum_i \theta_i(t)$ was $|\gamma| < 3 \times 10^{-4}$ rad and the body wave phase velocity $\omega(t) = -\frac{1}{2\pi}\frac{d}{dt}\left[\tan^{-1}(a_2(t)/a_1(t))\right]$ (2) was $\omega < -0.2\,\text{cycles}\,\text{s}^{-1}$ for at least 0.5 s. Ventral and dorsal turns were identified as segments where the overall body curvature was either $\gamma < -3.5 \times 10^{-4}$ rad or $\gamma > 3.5 \times 10^{-4}$ rad, respectively, for at least 0.5 s.

Resistive force theory simulations: We recover the rigid body motion from the tangent angle time series using linear resistive force theory, as in (11). We approximate the forces acting independently on each body segment as

$$\tilde{\mathbf{F}}_i(t) = \alpha_t \tilde{v}_i^t \hat{t} + \alpha_n \tilde{v}_i^n \hat{n}$$

where $\tilde{v}_i^{t,n}$ are the tangent and normal components of the velocity at each segment i, which can be written in terms of the velocity and displacements measured after subtracting the overall rigid body motion,

$$\tilde{\mathbf{v}}_i(t) = \mathbf{v}_i(t) + \tilde{\mathbf{V}}(t) + \tilde{\mathbf{\Omega}}(t) \times \Delta\mathbf{x}_i(t).$$

Then, by imposing a zero net-force and net-torque condition at each frame,

$$\sum_i \tilde{\mathbf{F}}_i = 0$$

$$\sum_i \tilde{\mathbf{F}}_i \times \Delta\mathbf{x}_i = 0,$$

we obtain a system of linear equations that for a given $\alpha = \alpha_n/\alpha_t$ can be solved for the components of the worm's velocity $\tilde{\mathbf{V}}(t)$ and angular velocity $\tilde{\mathbf{\Omega}}(t)$ (11). From these we can integrate the path taken by the worm's body to obtain a reconstructed $\tilde{\mathbf{x}}_{\text{CM}}(t)$.

We optimize the single free parameter α by comparing the reconstructed trajectories with the real worm trajectories $\mathbf{x}_{\text{CM}}^{\text{data}}$, Fig. S5(a). In particular, we minimize the maximum distance between 100 s trajectories randomly sampled from the dataset $L(\alpha) = \max(\|\tilde{\mathbf{x}}_{\text{CM}}^{\alpha}(t) - \mathbf{x}_{\text{CM}}^{\text{data}}(t)\|_2)$, $t \in [t_0, t_0 + 100\,\text{s}]$. To minimize $L(\alpha)$ we use the Nelder-Mead algorithm through the `scipy.optimize` library of Scipy (10). The software to translate posture into path can be found in https://github.com/AntonioCCosta/markov_worm, and follows closely the implementation of (11).

Metastable states: Metastable states correspond to collections of short-time movements that typically follow each other in time to give rise to stereotyped sequences. Leveraging our previous work (5), we search for metastable states along the slowest mode of the reversibilized dynamics (12). As shown in (13), the second eigenvector ϕ_2 of a time-reversibilized transition matrix P_r provides an *optimal* subdivision of the state space into almost invariant sets. In practice, we use the ensemble of worms to estimate P_r as

$$P_r(\tau) = \frac{P(\tau) + P(-\tau)}{2}, \quad [2]$$

where,

$$P_{ij}(-\tau) = \frac{\pi_j P_{ji}(\tau)}{\pi_i}$$

is the stochastic matrix governing the time-reversal of the Markov chain. The first non-trivial ($\lambda < 1$) right eigenvector of P_r, ϕ_2, allows us to define macrostates as collections of microstates s_i,

$$S^+(\phi_2^c) \coloneqq \bigcup_{i:\phi_2 \geq \phi_2^c} s_i,\ S^-(\phi_2^c) \coloneqq \bigcup_{i:\phi_2 < \phi_2^c} s_i,$$

where ϕ_2^c is a threshold that is chosen to maximize the metastability of a set. We measure the metastability of each set S by estimating how much of the probability density remains in S after a time scale τ,

$$\chi_{\pi,\tau}(S) = \frac{\sum_{i,j\in S} \pi_i P_{ij}(\tau)}{\sum_{i\in S} \pi_i}.$$

To estimate the overall measure of metastability across both sets S^+ and S^-, we define

$$\chi(\phi_2^c) = \min\left\{\chi_{\pi,\tau^*}(S^+), \chi_{\pi,\tau^*}(S^+)\right\}. \quad [3]$$

which we maximize with respect to ϕ_2^c. Metastable states are then defined with respect to the sign of $\phi_2 - \phi_2^c$. See (5) for further details and applications to known dynamical systems. In Fig. S7 we show the overall coherence measure as a function of ϕ_2 for the worm data.

Operator-based state space subdivision: We leverage the notion of relatively coherent sets (14) to subdivide the state space. However, instead of subdividing both metastable state at each iteration k, we identify the state with the most measure S_k^* and build a new transition matrix only with partitions belonging to that state,

$$P_{S_k^*}(\tau) = p(s_j(t+\tau)|s_i(t)),\ i,j \in S_k^*.$$

From $P_{S_k^*}$ we proceed as before: we compute the stationary distribution of S_k^* through the first left eigenvector of $P_{S_k^*}$, π_i^*, build the corresponding reversibilized transition matrix P_{r,S_k^*} and identify relatively metastable states through its first non-trivial eigenvector by maximizing Eq. (3) where π_i and $P_{ij}(\tau)$ are replaced by their relative counterparts π_i^* and $P_{S_k^*}$.

Simulating posture-to-path within mesoscopic behavioral states: To generate a centroid trajectory within a given state, we construct a transition matrix among the partitions corresponding to each of the mesoscopic states identified in Fig. 2(c) using data from all worms. We then proceed as in Figs. 2,3 to generate both posture time series and centroid trajectories. We first generate a symbolic sequence by sampling states according to the corresponding transition probability matrix $\hat{s}_j(t+\tau) \sim P_S(s_j|\hat{s}_i(t))$, $i,j \in S$. From the symbolic sequence, we then sample a time series segment $\vec{a}_{t:t+K^*}$ within each sampled partition, and use resistive force theory to translate the resulting $\theta(t)$ time series into locomotion. In this way, we can simulate posture and centroid trajectories for *in silico* worms that are forced to remain within a particular mesoscopic behavioral state for an arbitrary amount of time.

Probability of finding food as a function of distance and behavioral state: We estimate the likelihood of finding food in a given radius r by estimating the fraction of the area within a disc of radius r covered by the worm's body during 100 s trajectories, taking the worm's width to be 5% of its length. We then normalize these area fractions by the total across states, obtaining the $p(\text{food}|r, \text{state})$ showed in Fig. 5(b).

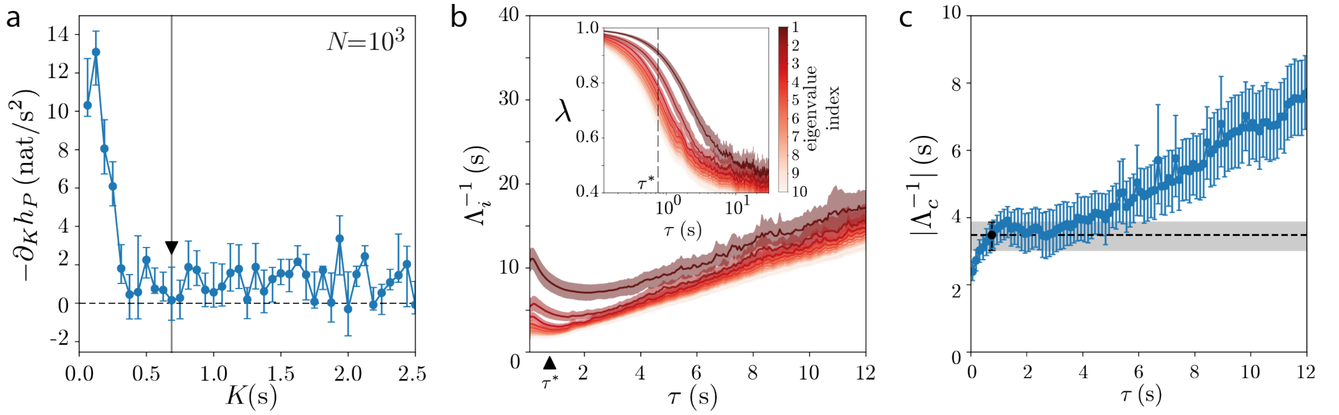

Fig. S1. Details of the Markovian approximation of the *C. elegans* posture dynamics through maximally predictive states. (a) - Change in short-time entropy rate as a function of delays K for $N = 1000$ partitions. The entropy rates reaches a plateau after $K \gtrsim 0.5$ s and we choose $K^* = 11$ frames $= 0.6875$ s. Error bars represent 95% confidence intervals bootstrapped across worms. (b) - The ten largest relaxation timescales of the reversibilized transition matrix as a function of transition time τ, and corresponding eigenvalues (inset). For $\tau \to \delta t$ the transition matrix is nearly the identity matrix (within τ most transitions occur within each partition), resulting in nearly degenerate eigenvalues close to 1 and an overestimation of the relaxation timescales of the reversibilized dynamics. On the other hand, when $\tau \gtrsim 5$ s the dynamics is mostly mixed, meaning that the transition matrix is composed of near copies of the steady-state distribution. In this regime, the eigenvalues of P_r, λ_i, become approximately constant, and therefore $\Lambda_i^{-1}(\tau) = -\tau/\log\lambda_i(\tau)$ grows linearly with τ. Between these two regimes, the relaxation timescales are approximately constant, and this robustness to τ is indicative of Markovian dynamics. We choose $\tau^* = 0.75$ s as the shortest τ^* consistent with Markovian dynamics. Error bars are 95% confidence intervals bootstrapped across worms. (c) - The reversibilized transition matrix provides an optimal partition into almost invariant sets (see Section **COARSE-GRAINING BEHAVIOR THROUGH ENSEMBLE DYNAMICS** for details), but the resulting kinetics does not necessarily capture the underlying dynamics. In fact, the obtained relaxation times are only an upper bound to the true relaxation timescales of the locally irreversible dynamics. To directly probe the Markovianity of the underlying slow dynamics, we estimate the relaxation times for the non-reversibilized coarse-grained transition matrix, which should not change with τ when the dynamics is Markovian. We approximate the slow relaxation dynamics by using the metastable states to build a two-state, coarse-grained Markov chain P_c, which necessarily has only real eigenvalues $\lambda_c \in \mathbb{R}$. The corresponding relaxation time is then obtained through $|\Lambda_c^{-1}| = -\tau/\log\lambda_c(\tau)$. In general, we find that the regime in which $|\hat{\Lambda}_2^{-1}|$ from P_r is constant (b) overlaps with regime in which $|\Lambda_c^{-1}|$ is also constant. In addition, while $|\Lambda_2^{-1}|(\tau^*)$ from P_r overestimates the expected $|\Lambda^{-1}|$ from "run" and "pirouette' transition rates, Fig. 4(b), the timescales obtained from P_c, $|\Lambda_c^{-1}|(\tau^*) = 3.48(3.03, 3.86)$ s are comparable to the ones estimated from the entire Markov chain in Fig. 2(a) and accurately predict the hopping dynamics. Error bars are 95% confidence intervals bootstrapped across worms.

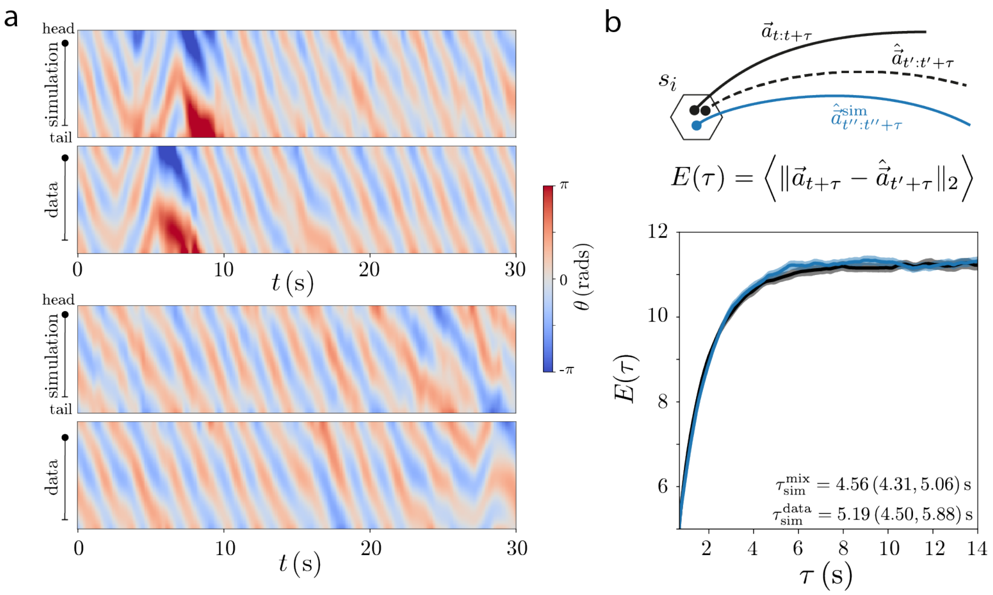

Fig. S2. Details of the Markov chain simulations. (a) - Two illustrative curvature vs. time plots comparing simulations with data. As expected due to the unpredictable nature of the dynamics (15), the quality of the predictions worsens as time progresses. Nonetheless, the structure of the dynamics is well preserved, making it hard to know *a priori* which of the two time traces is the data and which one is a simulation. (b) - We assess the predictive power of the Markov model by estimating how prediction errors grow over time. We define $E(\tau)$ by estimating the average distance between two trajectories starting within the same partition: t is chosen at random and $t' \neq t$ chosen such that $\hat{\vec{a}}_{t'}$ belongs to the same partition as $\hat{\vec{a}}_t$; the expectation value is then taken over multiple samples of t. We compare $E(\tau)$ estimated from simulations (blue) against sampling a trajectory from the data starting from the same partition (black). As summary statistics, we compute the time it takes before predictions completely mix, obtaining $\tau^{\mathrm{mix}}_{\mathrm{data}} = 5.19\,(4.50, 5.88)$ s for the data, just slightly higher than $\tau^{\mathrm{mix}}_{\mathrm{sim}} = 4.56\,(4.31, 5.06)$ s from simulations. In practice, we estimate the average distance between two randomly sampled points $e_\infty = \left\langle \|\vec{a}_i(t) - \vec{a}_i(t')\|_2 \right\rangle_{t,t'}$, and find the time it takes for $E(\tau) \leq 0.95 e_\infty$.

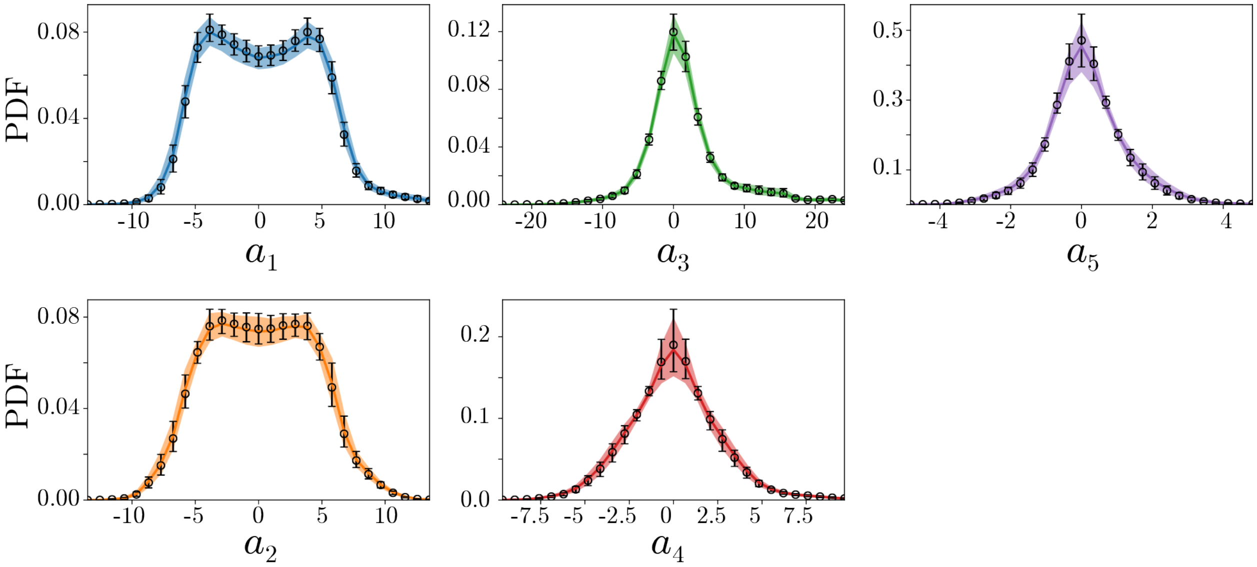

Fig. S3. Probability Density Function (PDF) of the "eigenworm" coefficients show a tight agreement between the data (colors) and simulations (black error bars), indicating that the inferred dynamics capture the steady-state distribution. Error bars and shaded areas correspond to 95% confidence intervals bootstrapped across worms.

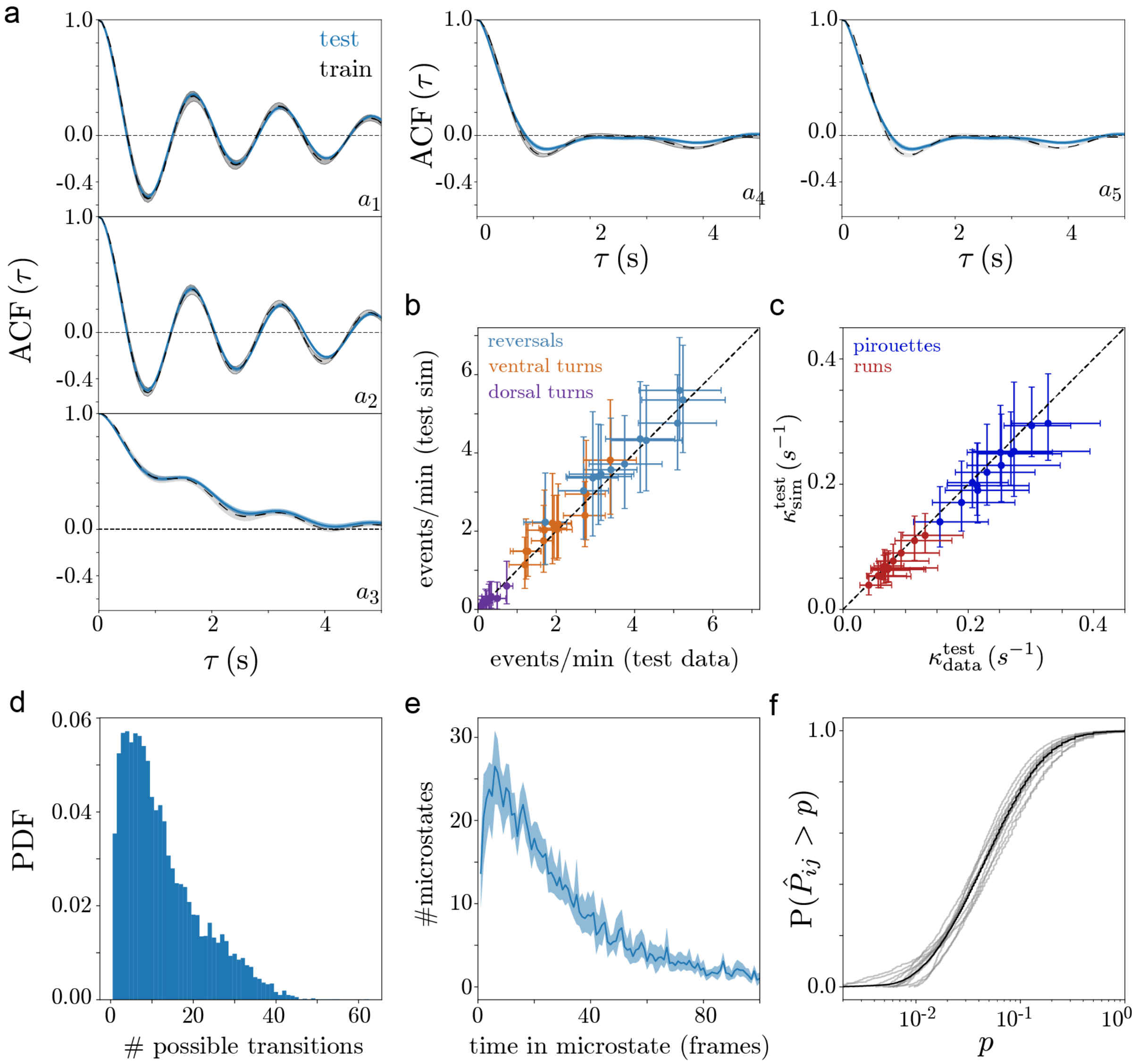

Fig. S4. **Cross-validation of the Markov model and details of the transition matrix estimation.** In Fig. 1 we build a Markov model with $N^* = 1000$ microstates to yield maximal expressivity before finite-size effects (which result in an underestimation of the entropy rate at large N) become evident. Here, to showcase how this choice of N^* results in a robust and generalizable model for the posture dynamics of each worm, we perform a cross-validation experiment by splitting the data of each worm into randomly sampled 70-30 training-test sets, learn the P_{ij} matrix using only 70% of the data and predict on a 30% unseen test set. We split the data into 10 segments (of 3.5 minutes each), randomly choose 3 segments for a test set and the remaining 7 segments for a training set, and repeat this process over 50 random seeds (see Methods for details). (a) - Autocorrelation function of the "eigenworm" coefficient time series obtained from the test data (blue) and simulations of the test data obtained from a model inferred in a separate training dataset (black) for an example worm. Shaded areas represent 95% confidence intervals obtained by bootstrapping across 50 random resampling of test and training datasets. (b,c) - Cross-validation of the rate of different behavioral events (b) and the transition rates between run and pirouette states (c) for all worms. We plot them as in Figs. 2(c,d), except that now we compare test set data with simulations obtained with a model built from a separate training set. Error bars correspond to 95% confidence intervals bootstrapped across the sampled behavioral events in 50 random reshuffles of train-test sets. (d) - To further showcase the estimation procedure, we assess how well transitions can be sampled. Despite the large number of microstates ($N^* = 1000$), the number of possible transitions from each state is constrained by the fact that τ^* is much smaller than the mixing time, rendering the transitions local. Therefore, the transition matrix is extremely sparse, with most state visiting only ≈ 10 other states resulting in a total of $\approx 10,000$ entries in the transition matrix. (e) - Histogram of the time spent in a microstate. While some microstates are visited rarely, the vast majority is visited at least 30 times. Error bars are bootstrapped across individual worms. (f) - Cumulative distribution function of the non-zero entries to the transition matrix. Most of the measured transition probabilities are between $0.01 \lesssim P_{ij} \lesssim 0.5$. The black line represents the P_{ij} estimated from the ensemble of worms, while each gray line represents the P_{ij} for each individual worm.

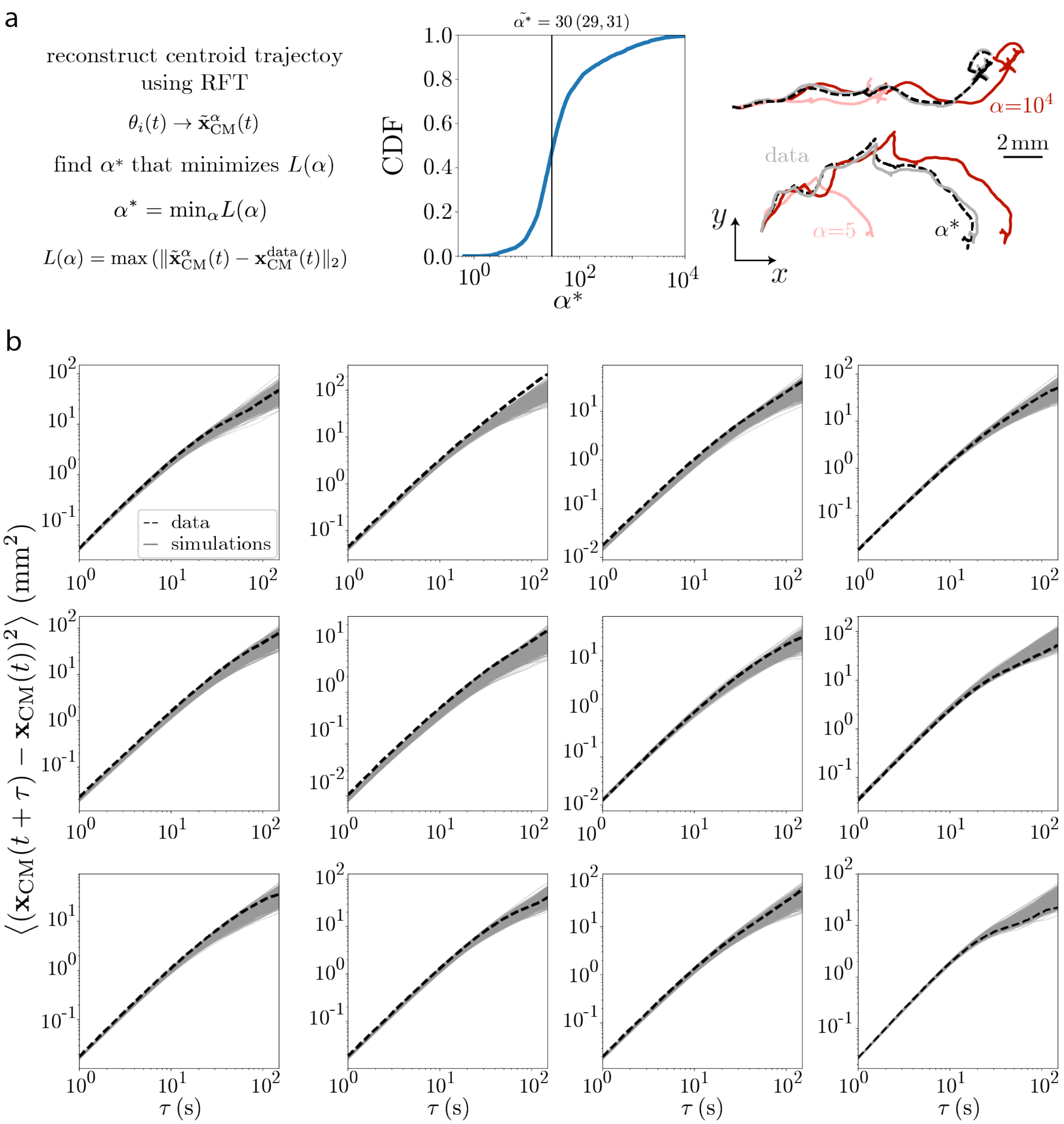

Fig. S5. Details of the posture-to-path simulations. (a) Optimizing the free parameter $\alpha = \alpha_n/\alpha_t$ in RFT (see Methods for details). We define a loss function $L(\alpha)$ as the maximum distance between the real worm trajectory and a reconstructed one, and sample randomly from 100 s segments. We the find the optimal α^* through the Nelder-Mead algorithm (see Methods). The resulting distribution of α^* is broad, as shown through the cumulative distribution function (CDF), with a median of $\tilde{\alpha^*} = 30\,(29, 31)$ s^{-1}. On the right, we display two example trajectory reconstructions for different values of α. For $\alpha = 5$ (comparable to experimental measures of (16)), RFT typically results in a substantial undershoot of the observed trajectories (as observed in (11)). For the no-slip condition, $\alpha \gg 1$, the resulting trajectories overshoot the real worm trajectories, indicating that some degree of slip is needed to accurately predict worm trajectories. With values of $\alpha^* \approx 30$ we get an accurate reconstruction of the worm trajectories. (b) Mean square displacements for the data (dashed line) of each worm, as well as for 1000 centroid trajectory simulations generated from symbolic sequences simulated with the Markov model (gray). By fitting a linear function in the interval $\tau \in [60, 100]$ s we obtained the effective diffusivity estimate of Fig. 4(b-right).

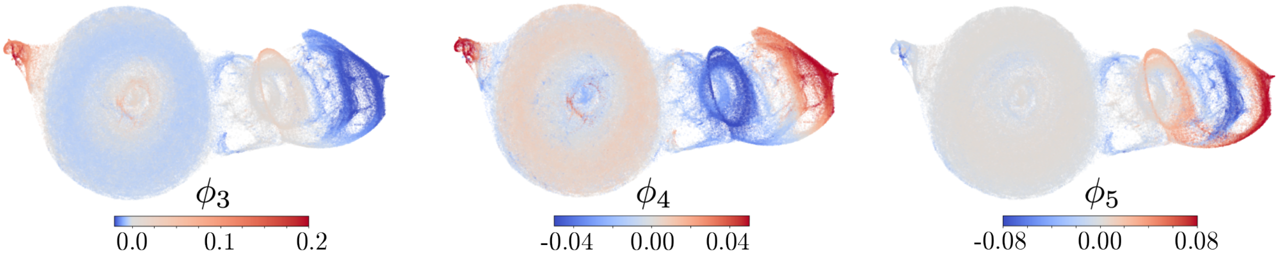

Fig. S6. Beyond "run and pirouette", additional slow modes of *C. elegans* behavior. While we have focused on ϕ_2, there is also important information in the remaining long-lived eigenfunctions. We color code the worm's maximally predictive state space by the projection along the 3 following eigenvectors, $\{\phi_3, \phi_4, \phi_5\}$, which are organized according to their relaxation times $\Lambda_3^{-1} > \Lambda_4^{-1} > \Lambda_5^{-1}$. We see that ϕ_3 differentiates dorsal and ventral turns, ϕ_4 differentiates turning and reversals, and ϕ_5 differentiates the compound motifs of shallow turns following a pause, from reversals that are followed by deep δ-turns. Together with ϕ_2 these modes provide a principled encoding of *C. elegans* off-food behavior across scales.

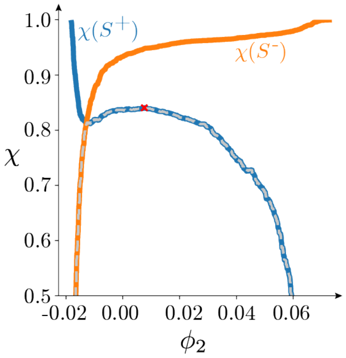

Fig. S7. Coherence measure used to define the metastable states. We define metastable states by maximizing the overall coherence, Eq. (3), of the two macroscopic states obtained by partitioning the state-space along ϕ_2 (see Methods for details). We here plot the coherence of each set (orange and blue), as well as the overall minima across sets χ, Eq. (3) (gray dashed line). The maximum of χ is highlighted with a red cross and indicates the value of ϕ_2 that defines the metastable states, $\phi_2 = \phi_2^c$.

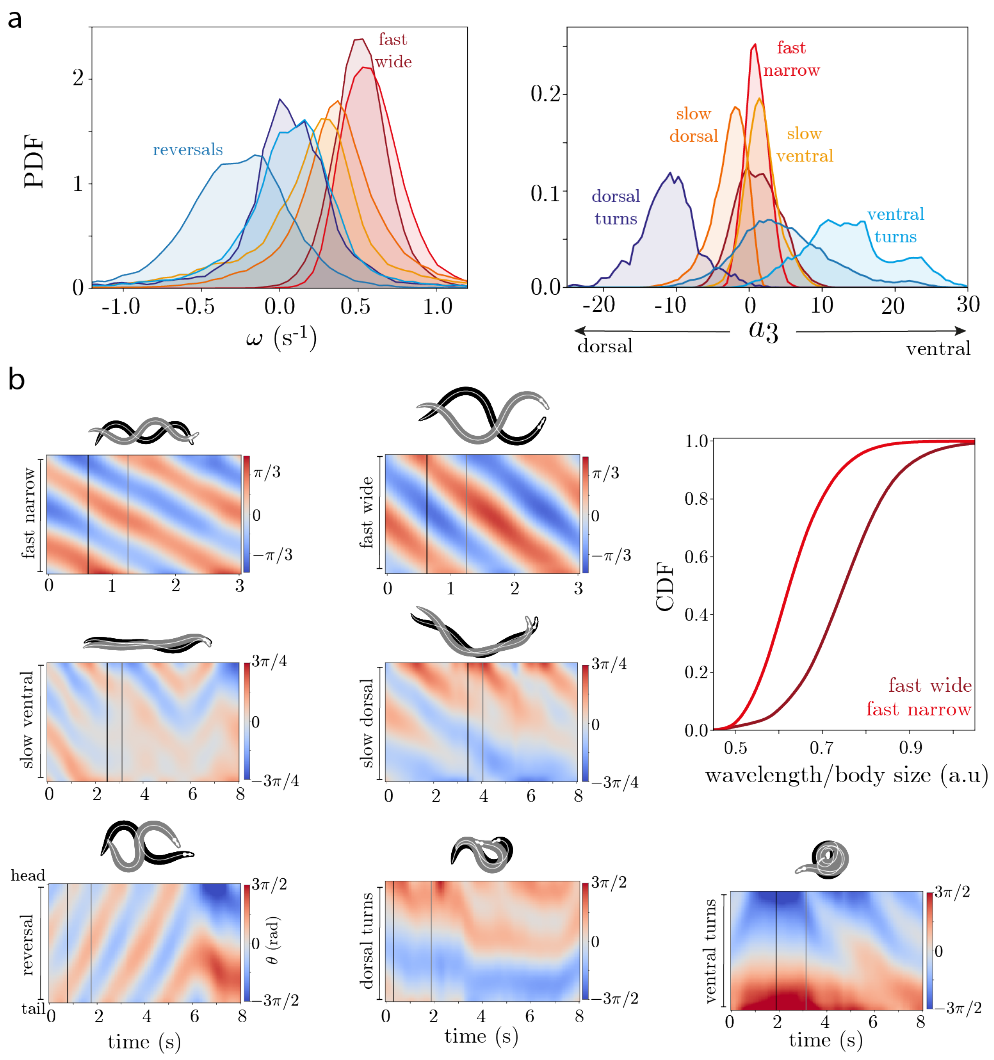

Fig. S8. Characterization of the 7 mesoscopic states obtained through a top-down subdivision of the *C. elegans* posture state space. (a) - Probability Distribution Function (PDF) of the body wave phase velocity $\omega = -\frac{1}{2\pi}\frac{d}{dt}\left(\tan^{-1}(a_2/a_1)\right)$ (left) and the dorso-ventral turning amplitude (right), as measured through the third "eigenworm" coefficient a_3 (2, 3). The state labels were given based on these probability distributions. (b) Example local tangent angles θ_i as a function of time in each of the states, as well as illustrative postures sampled at different time points (black and gray vertical lines). The two fast states (top) correspond to distinct gaits with different wavelengths (right), while the slow states (middle) lack a coherent body wave traveling from head to tail. Instead, the dorsally-biased slow states exhibits short timescale head-casting behavior (17), while the ventrally-biased state exhibits incoherent body motion with partial reversal and forward waves akin to a dwelling state (18).

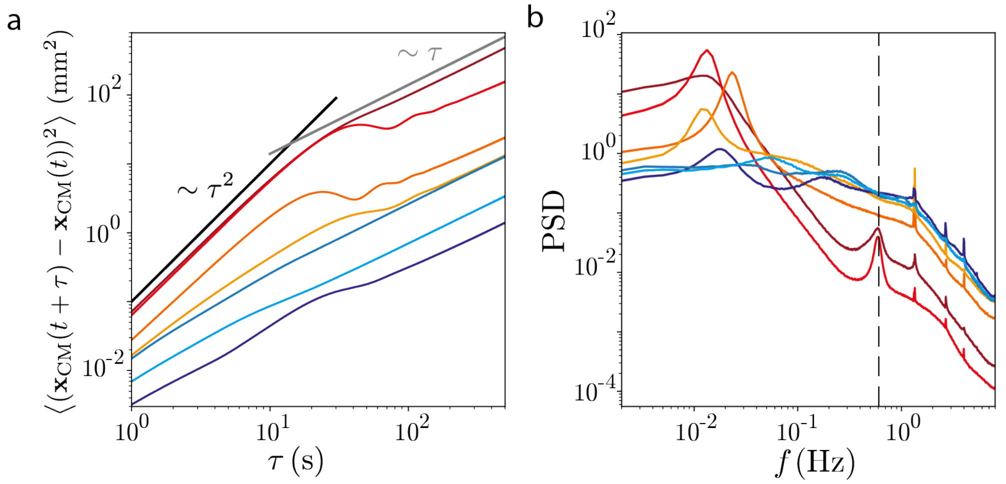

Fig. S9. Statistical properties of the trajectories simulated for each of the 7 mesoscopic states obtained through a top-down subdivision of the *C. elegans* posture state space. (a) - Mean square displacement (MSD) of the center-of-mass trajectories for each of the 7 states revealed in Fig. 4(c). Trajectories in the different states exhibit clearly distinct statistical properties: while "run" states generally exhibit a transition between super-diffusive behavior (MSD $\sim \tau^\beta,\ \beta > 1$) at short times and diffusive (or sub-diffusive) behavior (MSD $\sim \tau^\beta,\ \beta \leq 1$) at large times, the "pirouette"' states are mostly diffusive even at short times. In addition, some of the states exhibit non-trivial fluctuations in the MSD that result from the quasi-periodic loops observed in the trajectories shown in Fig. 5(a). (b) - Power spectral density of the velocity bearing angle η, $\vec{v}_{\mathrm{CM}} = v\cos(\eta)\,\hat{e}_x + v\sin(\eta)\,\hat{e}_y$, where $v = ||\vec{v}_{\mathrm{CM}}||$. Besides the fast oscillations due to the body wave dynamics (the vertical dashed line represents the body wave phase velocity in the fast wide run state), some states also exhibit low frequency peaks due to the loopy nature of the trajectories, which recur on a time scale orders of magnitude longer than the body wave period. The power spectral density was estimated using Welch's method (19) implemented through the `signal.welch` package from Scipy (10) with a Hann window and 10 min long trajectory segments. The error bars corresponding to 95% confidence bootstrapped across 1000 simulations for each state are too small to show.

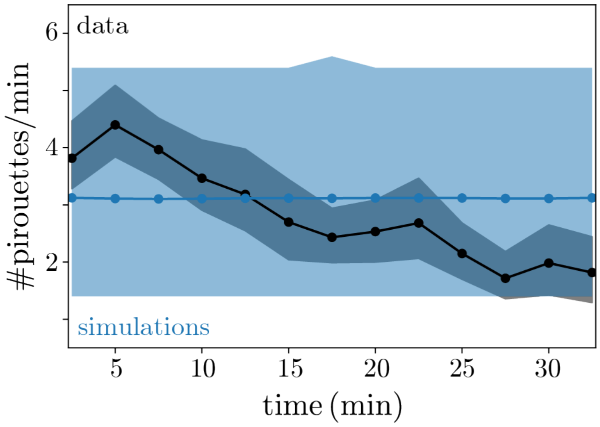

Fig. S10. The rate of pirouettes changes with time on the food-free plate, a non-stationary dynamics not captured in our Markov model. We estimate the rate of pirouettes as a function of time from the data (black), and find that it slowly decreases, reflecting a change in search strategy possibly as a result of updates to the animal's prior over the food distribution. Our simulations (blue) do not capture this slow change in the pirouette rate, as that would require an explicit time dependence in the transition probability matrix. Error bars correspond to 95% confidence bootstrapped across the 12 worms in our dataset. To estimate the rate of pirouettes, we coarse-grain the dynamics into "runs" and "pirouettes" as in Fig. 4, and find the number of pirouette events per minute in sliding 5 min windows with 2.5 min overlap. Since the sampling time of the Markov dynamics is τ^*, we discard pirouette events with a duration shorter than τ^* from this analysis.

Movie S1. (Download here) The simulated posture dynamics is virtually indistinguishable from the real worm data. We simulate a worm using the Markov model procedure of Fig. 2, and compare its posture dynamics with data starting from the same microstate. We show the curvature over time (left) for real (bottom) and simulated (top) worms, which are *a priori* indistinguishable from each other. We also show the corresponding RFT reconstructed skeletons (right), after subtracting the centroid position.

Movie S2. (Download here) Illustration of the posture to path simulations. We simulate a worm using the Markov model procedure of Fig. 2, and translate its posture dynamics into movement using the resistive force theory approach of Fig. 3.

References

1. AC Costa, T Ahamed, D Jordan, GJ Stephens, Dataset (https://doi.org/10.34740/KAGGLE/DS/3882219) (2023).
2. GJ Stephens, B Johnson-Kerner, W Bialek, WS Ryu, Dimensionality and dynamics in the behavior of C. elegans. *PLoS Comput. Biol.* **4**, e1000028 (2008).
3. OD Broekmans, JB Rodgers, WS Ryu, GJ Stephens, Resolving coiled shapes reveals new reorientation behaviors in C. elegans. *eLife* **5(e17227)** (2016).
4. JE Sulston, S Brenner, The DNA of Caenorhabditis elegans. *Genetics* **77**, 95–104 (1974).
5. AC Costa, T Ahamed, D Jordan, GJ Stephens, Maximally predictive states: From partial observations to long timescales. *Chaos: An Interdiscip. J. Nonlinear Sci.* **33**, 023136 (2023).
6. F Pedregosa, et al., Scikit-learn: Machine learning in Python. *J. Mach. Learn. Res.* **12**, 2825–2830 (2011).
7. EM Bollt, N Santitissadeekorn, *Applied and computational measurable dynamics.* (Society for Industrial and Applied Mathematics, Philadelphia, United States), (2013).
8. L McInnes, J Healy, J Melville, UMAP: Uniform Manifold Approximation and Projection for Dimension Reduction, arXiv [Preprint] (2018). https://arxiv.org/abs/1802.03426 (accessed 19 October 2023) (2018).
9. RB Lehoucq, DC Sorensen, C Yang, *ARPACK Users' Guide.* (Society for Industrial and Applied Mathematics), (1998).
10. E Jones, T Oliphant, P Peterson, et al., SciPy: Open source scientific tools for Python (2001–).
11. EE Keaveny, AEX Brown, Predicting path from undulations for c. elegans using linear and nonlinear resistive force theory. *Phys. Biol.* **14**, 025001 (2017).
12. M Dellnitz, O Junge, On the Approximation of Complicated Dynamical Behavior. *SIAM J. on Numer. Analysis* **36**, 491–515 (1999).
13. G Froyland, Statistically optimal almost-invariant sets. *Phys. D: Nonlinear Phenom.* **200**, 205–219 (2005).
14. T Ma, EM Bollt, Relatively coherent sets as a hierarchical partition method. *Int. J. Bifurc. Chaos* **23**, 1330026 (2013).
15. T Ahamed, AC Costa, GJ Stephens, Capturing the continuous complexity of behaviour in Caenorhabditis elegans. *Nat. Phys.* **17**, 275–283 (2021).
16. Y Rabets, M Backholm, K Dalnoki-Veress, WS Ryu, Direct measurements of drag forces in c. elegans crawling locomotion. *Biophys. journal* **107 8**, 1980–1987 (2014).
17. HS Kaplan, O Salazar Thula, N Khoss, M Zimmer, Nested neuronal dynamics orchestrate a behavioral hierarchy across timescales. *Neuron* **105**, 562–576.e9 (2020).
18. M Fujiwara, P Sengupta, SL McIntire, Regulation of body size and behavioral state of C. elegans by sensory perception and the EGL-4 cGMP-dependent protein kinase. *Neuron* **36**, 1091–102 (2002).
19. P Welch, The use of fast fourier transform for the estimation of power spectra: A method based on time averaging over short, modified periodograms. *IEEE Transactions on Audio Electroacoustics* **15**, 70–73 (1967).
